# Somatic Genetics Analysis of Sleep in Adult Mice

**DOI:** 10.1523/JNEUROSCI.0089-22.2022

**Published:** 2022-07-13

**Authors:** Guodong Wang, Qi Li, Junjie Xu, Shuai Zhao, Rui Zhou, Zhenkang Chen, Wentong Jiang, Xue Gao, Shuang Zhou, Zhiyu Chen, Quanzhi Sun, Chengyuan Ma, Lin Chen, Bihan Shi, Ying Guo, Haiyan Wang, Xia Wang, Huaiye Li, Tao Cai, Yibing Wang, Zhineng Chen, Fengchao Wang, Qinghua Liu

**Affiliations:** ^1^National Institute of Biological Sciences, Beijing, 102206, China; ^2^Tsinghua Institute of Multidisciplinary Biomedical Research, Tsinghua University, Beijing, 102206, China; ^3^Graduate School of Peking Union Medical College, Chinese Academy of Medical Sciences, Beijing, 100730, China; ^4^College of Life Sciences, Beijing Normal University, Beijing, 100875, China; ^5^Institute of Automation, Chinese Academy of Sciences, Beijing, 100080, China; ^6^College of Biological Sciences, China Agriculture University, Beijing, 100094, China; ^7^Children's Medical Center Research Institute, UT Southwestern Medical Center, Dallas, Texas 75235; ^8^Chinese Institute of Brain Science, Beijing, 102206, China; ^9^International Institute for Integrative Sleep Medicine, University of Tsukuba, Tsukuba, 305-8575, Japan

**Keywords:** AAV-PHP.eB, adult brain chimeric, CRISPR/Cas9, KO, sleep phenotypes, somatic genetics analysis

## Abstract

Classical forward and reverse mouse genetics require germline mutations and, thus, are unwieldy to study sleep functions of essential genes or redundant pathways. It is also time-consuming to conduct EEG/EMG-based mouse sleep screening because of labor-intensive surgeries and genetic crosses. Here, we describe a highly accurate SleepV (video) system and adeno-associated virus (AAV)-based adult brain chimeric (ABC)-expression/KO platform for somatic genetics analysis of sleep in adult male or female mice. A pilot ABC screen identifies CREB and CRTC1, of which constitutive or inducible expression significantly reduces quantity and/or quality of non-rapid eye movement sleep. Whereas ABC-KO of exon 13 of *Sik3* by AAV-Cre injection in *Sik3-E13^flox/flox^* adult mice phenocopies *Sleepy (Sik3*^*Slp*/+^*)* mice, ABC-CRISPR of *Slp/Sik3* reverses hypersomnia of *Sleepy* mice, indicating a direct role of SLP/SIK3 kinase in sleep regulation. Multiplex ABC-CRISPR of both orexin/hypocretin receptors causes narcolepsy episodes, enabling one-step analysis of redundant genes in adult mice. Therefore, this somatic genetics approach should facilitate high-throughput analysis of sleep regulatory genes, especially for essential or redundant genes, in adult mice by skipping mouse development and minimizing genetic crosses.

**SIGNIFICANCE STATEMENT** The molecular mechanisms of mammalian sleep regulation remain unclear. Classical germline mouse genetics are unwieldy to study sleep functions of essential genes or redundant pathways. The EEG/EMG-based mouse sleep screening is time-consuming because of labor-intensive surgeries and lengthy genetic crosses. To overcome these “bottlenecks,” we developed a highly accurate video-based sleep analysis system and adeno-associated virus-mediated ABC-expression/KO platform for somatic genetics analysis of sleep in adult mice. These methodologies facilitate rapid identification of sleep regulatory genes, but also efficient mechanistic studies of the molecular pathways of sleep regulation in mice.

## Introduction

Despite significant advance in understanding the neural pathways that control executive sleep/wake switching (Saper et al., 2005, 2010; Weber and Dan, 2016; Scammell et al., 2017; Liu and Dan, 2019), the molecular mechanisms of mammalian sleep regulation are largely unclear (Sehgal and Mignot, 2011; Allada et al., 2017; Webb and Fu, 2021). It remains to be identified which genes constitute the core sleep regulatory pathways, and where these molecular pathways may function in the mouse brain. However, it is very costly and time-consuming to conduct large-scale mouse sleep screening for two main reasons: (1) both forward and reverse genetics approaches require germline mutations and genetic crosses (3 months/cross); and (2) the EEG/EMG-based sleep analysis requires labor-intensive and invasive surgeries.

Accumulating evidence suggests that sleep is essential for survival in invertebrate and vertebrate animals (Rechtschaffen et al., 1989; Bentivoglio and Grassi-Zucconi, 1997; Shaw et al., 2002; Vaccaro et al., 2020). Thus, the core sleep regulatory genes may be essential for survival in mice. Conditional KO mice are commonly used to bypass early lethality and analyze the temporal and/or tissue-specific functions of essential genes (Gierut et al., 2014). Typically, the Cre/loxP-mediated site-specific recombination is used to exercise the critical exon(s) and disrupt the target gene in a tissue-specific and/or temporal manner. However, this time-consuming (1-2 years) approach requires construction of appropriate Cre transgenic and flox mouse strains, but also multiple genetic crosses to generate sufficient number of conditional KO mice for comprehensive sleep analysis (Gierut et al., 2014).

Given the vital importance of sleep in physiology and survival, it is likely that redundant pathways exist for sleep regulation. Thus, ablation of a gene of interest may not cause significant sleep phenotype owing to genetic redundancy. Additionally, mice with germline mutations may adapt or compensate for sleep phenotype before the EEG/EMG-based sleep analysis that is normally conducted in adult mice. Moreover, it is a costly and tedious process to generate double or triple KO mice by traditional germline genetics methods. A combination of triple-target CRISPR/Cas9 and modified embryonic stem cell technologies facilitates biallelic KO of redundant genes for sleep phenotype analysis in a single generation, but it does not work for essential genes (Sunagawa et al., 2016; Susaki et al., 2017).

Recombinant adeno-associated viruses (AAVs) have been widely used as vehicles for gene expression, knockdown/KO, and gene therapy in the CNS (Borel et al., 2014; Suzuki et al., 2016; Yang et al., 2016). To spatially restrict gene expression, these applications often require local AAV injection into specific regions of the mouse brain (Hunker et al., 2020; Zell et al., 2020; Castro et al., 2021). Alternatively, intravenous administration of AAVs provides a noninvasive strategy for systemic gene delivery into the CNS (Foust et al., 2009; Choudhury et al., 2016; Deverman et al., 2016). In particular, Gradinaru and colleagues have used the Cre-recombination-based AAV-targeted evolution (CREATE) to isolate AAV9 variants, such as AAV-PHP.B and AAV-PHP.eB, which can efficiently bypass the blood–brain barrier and transduce the majority of adult brain neurons and astrocytes in certain mouse strains (Deverman et al., 2016; Chan et al., 2017). Both AAV-PHP.B and AAV-PHP.eB have been successfully used to deliver systemic gene expression (Luoni et al., 2020; Silva-Pinheiro et al., 2020; Liu et al., 2021), or target gene ablation by CRISPR/Cas9 across the adult mouse brain neurons (Yamaguchi and de Lecea, 2019; Torregrosa et al., 2021; Xiao et al., 2021).

Here, we systematically assembled, optimized and used this AAV-PHP.eB-based adult brain chimeric (ABC)-expression/KO platform for somatic genetics analysis of sleep in adult mice. We also developed a highly accurate, noninvasive, and fully automated SleepV (video) system for high-throughput mouse sleep screening. This somatic genetics approach, coupled with SleepV system, should facilitate high-throughput screening of new sleep regulatory genes, especially from essential or redundant genes, in adult mice by skipping the mouse development and minimizing genetic crosses.

## Materials and Methods

### Mice

All animal experiments were performed according to procedures approved by the institutional Animal Care and Use Committee of National Institute of Biological Sciences, Beijing (NIBS). All mice were provided food and water *ad libitum* and were housed under humidity- and temperature-controlled conditions (22°C-24°C) on a 12 h light/12 h dark cycle. *Rosa26^Cas9^* (JAX, 026179) and *Rosa26^LSL-Cas9^* (JAX, 026175) were purchased from the The Jackson Laboratory. The *Creb1^flox^* and *Sik3-E13^flox^* mice were generated by flanking exon 4 of *creb1* or exon13 of *Sik3* with two loxP sites, respectively, in the transgenic animal facility at NIBS. Littermates were used for experiments whenever possible. Most experiments used male mice, except for *Sik3-E13^flox^;Rosa26^Cas9^* female mice when specifically indicated. The age of mice ranged from 11 to 18 weeks.

### Generation of AAV vectors

The pAAV- EF1α-DIO-H2B-eGFP plasmid was a gift from Minmin Luo's laboratory (NIBS). pAAV-CBh-Cre was constructed by cloning the CBh-Cre cassette from pAAV-U6-sgRNA(backbone)-CBh-Cre (Addgene, 60 229) into MluI-HF (R3198L, NEB) and HindIII-HF (R3104S, NEB) digested pAAV-hSyn-eGFP (Addgene, 105539). pAAV-hSyn-Cre was subcloned with AsiSI (R0630L, NEB) and MluI-HF (R3198L, NEB) restricted endonuclease from pAAV-hSyn-eGFP by replacing eGFP with Cre.

The cDNAs of Arc (CCDS37102.1), Homer1a (CCDS26687.1), Rab3a (CCDS22379.1), Plk2-dn (CCDS26767.1, 332-682aa), CaMKIIα (CCDS29276.1), CaMKIIβ (CCDS24411.1), CaMKIV (CCDS29122.1), CDK5 (CCDS51434.1), CREB (CCDS15005.1), and CRTC1 (CCDS40372.1) were amplified from mouse brain cDNA library by primers contain AgeI-HF (R3552L, NEB) and BamHI-HF (R3136L, NEB) digested site. CaMKIV-dn and CRTC1^CA^ were constructed from CaMKIV or CRTC1 by site-directed mutagenesis using specific primers that contain K75E or S151A/S245A mutations. MEF2^VP16^ and CREB^VP16^ were constructed by fusing MEF2C (CCDS84042.1, 1-117aa) and CREB (CCDS15004.1, 88-341aa) with C-terminal VP16-3xHA and N-terminal VP16 by Gibson assembly. All these cDNAs were inserted into pAAV-hSyn-eGFP by replacing eGFP with individual cDNA.

The sgRNA sequences (Table 1) were designed based on the Mouse GeCKO v2 Library (Sanjana et al., 2014; Sunagawa et al., 2016). Annealed oligos for individual sgRNA were first inserted into the SapI (R0569S, NEB) digested pAAV-U6-sgRNA(backbone)-hSyn-Cre-2A-EGFP-KASH (Addgene, 60231) vector. To better construct the double and triple sgRNA vectors, we had inserted a multiple cloning site sequence before the hSyn promoter of pAAV-hSyn-eGFP and pAAV-hSyn-Cre. Then, these modified pAAV-hSyn-eGFP or pAAV-hSyn-Cre plasmids containing double or triple U6:sgRNA cistrons were constructed through PCR amplification of two or three different sgRNAs that target the same gene by two or three pairs of primers, Restriction endonuclease of MluI-HF (R3198L, NEB), BamHI-HF (R3136L, NEB), XbaI (R0145S, NEB), and KpnI-HF (R3142S, NEB) were used for the vectors and fragments digestion, Quick Ligation Kit (M2200L, NEB) was used for the ligation of these appropriate clones. All these plasmids were saved and amplified in NEB Stable Competent *Escherichia coli* (C3040I, NEB).

**Table 1.** Primer sequences

	Forward (F)/reverse (R)	Sequence
CBh-Cre	F	cgacgcgtACCCGTTACATAACTTACGGTAAATGGC
	R	cccaagcttGATATCGAATTCTTAAGCGTAATCTG
Arc	F	atctgataccggtgccaccATGGAGCTGGACCATATGACCAC
	R	atctgatggatccTTCAGGCTGGGTCCTGTCACTG
Homer1a	F	atctgataccggtgccaccATGGGGGAGCAACCTATCTTCAGC
	R	atctgatggatccacgcgtCTTAATCATGATTGCTGAATTGAATGTGTACC
CRTC1	F	atctgataccggtgccaccATGGCGACTTCGAACAATCC
	R	atctgatggatccCAGGCGGTCCATTCGGAAGG
CRTC1-S151A	F	GGAGGACCAACgCTGACTCTGC
	R	GCAGAGTCAGcGTTGGTCCTCC
CRTC1-S245A	F	CACAGGGGGCgCCCTTCCTGAC
	R	GTCAGGAAGGGcGCCCCCTGTG
CREB	F	agctaccggtgccaccATGACCATGGAATCTGGAGCAG
	R	cgcggatccATCTGATTTGTGGCAGTAAAGGTCC
CREB-S119A	F	GGAGGCCTgCCTACAGGAAAATTTTGAATGAC
	R	CTGTAGGcAGGCCTCCTTGAAAGGATTTCC
Rab3a	F	atctgataccggtgccaccATGGCTTCCGCCACAGACTC
	R	atctgatggatccGCAGGCACAATCCTGATGAG
Plk2-dn	F	atctgataccggtgccaccATGCTGCAGGGTTTCACTCCGGAC
	R	atctgatggatccGTTACATCTCTGTAAGAGCATGTTC
CaMKIIα	F	atctgataccggtgccaccATGGCTACCATCACCTGCACC
	R	atctgatggatccATGCGGCAGGACGGAGGGCG
CaMKIIβ	F	atctgataccggtgccaccATGGCCACCACGGTGACCTG
	R	atctgatggatccCTGCAGCGGGGCCACTGGAG
CaMKIV	F	atctgataccggtgccaccATGCTCAAAGTCACGGTGCCCTC
	R	atctgatacgcgtGTACTCTGGCTGAATCGCATCC
CaMKIV-dn	F	CAAAGTGTTAAAGgAAACAGTGGACAAG
	R	CTTGTCCACTGTTTcCTTTAACACTTTG
MEF2C	F	atctgataccggtgccaccATGGGGAGAAAAAAGATTCAGATTACG
	R	atctgatggatccTGTTGCCCATCCTTCAGAGAGTC
CDK5	F	atctgataccggtgccaccATGCAGAAATACGAGAAACTGGAG
	R	atctgatggatccTGGGGGACAGAAGTCAGAGAAG
TRE3G	F	gcagggcccGAGTTTACTCCCTATCAGTGATAGAGAACGTATGAAGAGT
	R	gcaaccggtTTTACGAGGGTAGGAAGTGGTACGGAAAGTTGGT
Set1 sgRNA for nontarget control	sgRNA1-F	accACGTGTAAGGCGAACGCCTT
	sgRNA1-R	aacAAGGCGTTCGCCTTACACGT
	sgRNA2-F	accGACTCCGGGTACTAAATGTC
	sgRNA2-R	aacGACATTTAGTACCCGGAGTC
	sgRNA3-F	accCCGCGCCGTTAGGGAACGAG
	sgRNA3-R	aacCTCGTTCCCTAACGGCGCGG
Set2 sgRNA for nontarget control	sgRNA1-F	accGCGAGGTATTCGGCTCCGCG
	sgRNA1-R	aacCGCGGAGCCGAATACCTCGC
	sgRNA2-F	accGCTTTCACGGAGGTTCGACG
	sgRNA2-R	aacCGTCGAACCTCCGTGAAAGC
	sgRNA3-F	accATGTTGCAGTTCGGCTCGAT
	sgRNA3-R	aacATCGAGCCGAACTGCAACAT
sgRNA for NeuN	sgRNA1-F	accTCGGGGTCCCTGAACCGGAA
	sgRNA1-R	aacTTCCGGTTCAGGGACCCCGA
	sgRNA2-F	accGCTCAGATGCTGACCGAGCC
	sgRNA2-R	aacGGCTCGGTCAGCATCTGAGC
	sgRNA3-F	accGCTGAATGGGACGATCGTAG
	sgRNA3-R	aacCTACGATCGTCCCATTCAGC
Set1 sgRNA for SIK3	sgRNA1-F	accACAGACCACTTGGTAGCCCA
	sgRNA1-R	aacTGGGCTACCAAGTGGTCTGT
	sgRNA2-F	accACTCACCCATATGTCCACTT
	sgRNA2-R	aacAAGTGGACATATGGGTGAGT
	sgRNA3-F	accGTTCAAACAGATCGTCACAG
	sgRNA3-R	aacCTGTGACGATCTGTTTGAAC
Set2 sgRNA for SIK3	sgRNA1-F	accTGACAGAATACGCTAGCGGA
	sgRNA1-R	aacTCCGCTAGCGTATTCTGTCA
	sgRNA2-F	accGCCGCCCCAGAGCTCTTCGA
	sgRNA2-R	aacTCGAAGAGCTCTGGGGCGGC
	sgRNA3-F	accGACAGAGCGCATGATTTACC
	sgRNA3-R	aacGGTAAATCATGCGCTCTGTC
sgRNA for OXR1	sgRNA1-F	accATCACCGAGTCGTGGCTCTT
	sgRNA1-R	aacAAGAGCCACGACTCGGTGAT
	sgRNA2-F	accCTGAGCTAGCCAATCGCACC
	sgRNA2-R	aacGGTGCGATTGGCTAGCTCAG
	sgRNA3-F	accAGCACAGCTCGCCGTGCCCG
	sgRNA3-R	aacCGGGCACGGCGAGCTGTGCT
sgRNA for OXR2	sgRNA1-F	accTAAAGCAGATCCGAGCACGA
	sgRNA1-R	aacTCGTGCTCGGATCTGCTTTA
	sgRNA2-F	accGATGCTGTTTCGAGCCCGTT
	sgRNA2-R	aacAACGGGCTCGAAACAGCATC
	sgRNA3-F	accAATGGCGTACCATCGGTCCA
	sgRNA3-R	aacTGGACCGATGGTACGCCATT
3xsgRNA- multiple cloning site	F	cgcgggtaccctcgagtctagattaattaaggcgcgccggatccacgcgtggcc
	R	acgcgtggatccggcgcgccttaattaatctagactcgagggtacc
sgRNA1	F	atcgACGCGTGAGGGCCTATTTCC
	R	cgcggatcCACGCGCTACGGACTAGC
sgRNA2	F	cgggatccGAGGGCCTATTTCCCATGATTCC
	R	gctctagaCACGCGCTACGGACTAGC
sgRNA3	F	gctctagaGAGGGCCTATTTCCCATGATTCC
	R	ggggtaccCACGCGCTACGGACTAGC
qRT-PCR for OXR1	F	ATTCCGGGAGCAGTTCAAGG
	R	GCTCTGCAAGGACAAGGACT
qRT-PCR for OXR2	F	CCACGGACTATGACGACGAG
	R	TTCCCGATGAGAGCCACAAC
qRT-PCR for Arc	F	ATGAATGGGCCAGCCAAGAA
	R	AATGGCTTCACGGGAGAGTG
qRT-PCR for CDK5	F	CCCAGCTACAACATCCTTGGT
	R	CGCTGCACAGGGTTACACTT
qRT-PCR for CREB	F	AACCAGCAGAGTGGAGATGC
	R	GATGTTGCATGAGCTGCTGG
qRT-PCR for Homer1a	F	GACGATGAGAGAACACCCGA
	R	GATTGCTGAATTGAATGTGTACCT
qRT-PCR for CRTC1	F	TGCCCAACGTGAACCAGATT
	R	CATGATGTCGTGTGGTCCGA
qRT-PCR for GAPDH	F	TTCACCACCATGGAGAAGGC
	R	CTCGTGGTTCACACCCATCA

Uppercase letters are used for the sequence of the gene. Lowercase letters are used for the added sequence and mutation site. Underlines indicate the restriction site.

### Virus generation

AAV-PHP.eB viruses were packaged in AAVpro 293T cells (Clontech, 632273). Cells were harvested by cell lifter (Biologix, 70-2180) at 72 h after cotransfection with PHP.eB (Addgene, 103005), pAdDeltaF6 (Addgene, 112867) and transfer plasmids using polyethylenimine MAX (Polysciences, 24 765). Cell pellets were suspended in 1× gradient buffer (10 mm Tris-HCl, pH 7.6, 150 mm NaCl, 10 mm MgCl_2_). Cells were lysed by five repeated cycles of freezing in liquid nitrogen for 7 min, thawing in 37°C water bath for 3 min, and vortexing for 2 min. Cell lysate was mixed with ≥50 U/ml of Benzonase nuclease (Millipore, E1014) and incubated at 37°C for 30 min. After centrifugation at 20,000 × *g* for 30 min at 4°C, the supernatant was transferred to a iodixanol (Optiprep, D1556) step gradient (15%, 25%, 40%, and 58%) for ultracentrifugation. After centrifugation at 40,000 rpm for 4 h at 4°C, virus particles were collected from the layer of 40% iodixanol gradient using a sterile syringe. Purified AAVs were concentrated using Amicon filters (EMD, UFC801096) and formulated in sterile PBS supplemented with 0.01% Pluronic F68 (Invitrogen, 24040032). Virus titers were determined by qPCR using a linearized AAV plasmid as a standard.

### Video-based sleep recording

For video-based sleep screening, 9- to 11-week-old C57BL/6J male mice were retro-orbitally injected with 10^12^ AAV-PHP.eB viruses expressing different cDNAs from the hSyn promoter. Two weeks after virus injection, mice were individually housed in Ancare cages with food and water provided *ad libitum* on a 12 h light/12 h dark cycle. The sleep/wake behaviors of the mice were recorded by an infrared camera (704 × 576 resolution) at 25 frames/s. Two infrared LED light strips were placed above the cages for videotaping mouse behaviors during the dark phase. Cameras and infrared LED lights were mounted 50 cm above the cages. Infrared LED lights were installed on both side of the camera, and were 28.5 cm away from the camera. For all mice, 3 d of video data were continuously recorded to calculate average daily sleep time for each mouse.

### Automatic sleep/wake staging by SleepV

The automatic sleep/wake staging of video data consists of two stages: (1) an artificial intelligence-augmented video analysis algorithm was developed to extract various information of test mouse from each sampled frame; and (2) the state of test mouse (active or inactive) at the video clip level was obtained by grouping the frame-level information within a time window.

#### Video analysis stage

The suspected ROIs are first extracted from each sample frame using traditional image processing techniques, such as Gaussian filtering, adaptive and global threshold. More than one ROI is allowed to be detected because of the complication of food, water gel, and shadows caused by environmental light. This multidetection strategy ensures that the true mouse region is not missed. A deep neural network is then used to make a binary classification of each ROI as a mouse or not. Our neural network backbone was based on Resnet18 architecture (He et al., 2016). We trained the Resnet18 model using 7200 positive images and 9000 negative images with manual labeling. All image frames for training were randomly chosen from 6 mice, which covered both light and dark conditions, and as many mouse postures as possible. All the experiments were conducted on a workstation with a NVIDIA Titan XP GPU, Intel Core i7-7000 CPU.

During the training process, labeled frames data were randomly split into two parts: 80% for training and 20% for validation. We chose cross entropy as the binary classification loss function, and Stochastic Gradient Descent as the optimizer with a fixed learning rate of 0.001. The training process lasted 50 epochs; as a result, the model accuracy was 99.3% on training dataset and ∼96.1% on validation dataset. The ROI with the highest confidence score is designated as the mouse region, while the others were classified as the background noise.

According to the size of ROI and position coordinate, we extracted several descriptive features, that is, the network prediction score (predict), the detected mouse mask area (IoU), the center of mass of the mask (activity), and the gray information (color) within the detected mask. Similarly, the frame-level features within a time window centered on that frame were also extracted. Denote f_{t} as the current frame, {f{t-m}, f{t-m + 1}, … f{t + m}} refer to the frames within this time window, where m is a hyperparameter. The differences between f_{t} and f{t + i}, including predict score difference P_i (predict), mask difference M_i (IoU), center of mass difference of the mouse body A_i (activity), and gray pixel difference G_i (color), were calculated and normalized. We defined P_i > 0.1, or G_i > 3, or A_i > 5, or the normalized value (G_i + M_i + P_i)/3 > 0.5 as the threshold to judge the mouse was active at frame t.

#### Sleep/wake state determination stage

According to the frame-level preliminary judgment, SleepV defines the sleep state as ≥40 s of continuous immobility as previously developed video-based sleep analysis software (Pack et al., 2007; Fisher et al., 2012). To further improve the performance of SleepV, we used a series of 0, 5, 10, 15, 20, 25, and 30 s thresholds to filter subtle mouse movements during sleep and compared the sensitivity, specificity, accuracy, and sleep bouts number of SleepV with EEG/EMG analysis. We found that annotating ≤15 s of mouse movements in between two sleep states as sleep achieves the best performance of automatic sleep/wake staging by SleepV, which is comparable to that of semiautomatic sleep/wake staging by EEG/EMG analysis software with manual corrections. SleepV code was deposited in GitHub (https://github.com/wochiguodong/SleepV.git).

#### Tet-on inducible ABC system

For Tet-on inducible system, pAAV-TRE-eGFP, pAAV-TRE-CREB^VP16^, and pAAV-TRE-CRTC1^CA^ were constructed by replacing hSyn promoter with tetracycline response element (TRE) in pAAV-hSyn-eGFP, pAAV-hSyn-CREB^VP16^, and pAAV-hSyn-CRTC1^CA^. The pAAV-EF1α-rTTA was constructed by placing reverse tetracycline-controlled transactivator (rtTA) under the control of the EF1α promoter. After 1 week recovery after EEG/EMG surgery, mice were coinjected retro-orbitally with AAV-EF1α-rTTA and AAV-TRE-eGFP, AAV-TRE-CREB^VP16^, or AAV-TRE-CRTC1^CA^. Two weeks after AAV injection, these mice were subjected to continuous EEG/EMG recording for 3 d with doxycycline (Dox)-containing (1 mg/ml) drinking water.

#### Genomic DNA extraction and captured Illumina sequencing

Genomic DNA was extracted from mouse brain using TIANamp Genomic DNA Kit (Tiangen, DP304) following the recommended protocol. Genomic DNA (1-1.5 g) was sheared to 300-400 bp by Covaris S220 (Covaris) and purified with 1× magnetic beads (Ampure XP; Beckman Coulter). Sheared DNA fragments were subjected to Illumina paired-end DNA library using NEBNext ultra II DNA Library Prep Kit (E7645L, NEB). Preparation and PCR-amplified for three cycles and libraries size were selected with 0.55-1× magnetic beads (Ampure XP, Beckman). Amplified libraries were sequenced using the HiSeq X ten Platform (Illumina) as paired-end 150 base reads according to the manufacturer's protocol.

Illumina raw sequencing reads were processed through a standard pipeline consisting of low-quality read filtering through Trimmomatic (version 0.36), alignment to mouse genome GRCm38 (mm10) using the Burrows–Wheeler Aligner (version 0.7.17-r1188) algorithm. The aligned BAM files were processed using the Genome Analysis Toolkit (version 4.1.4), including mark PCR duplicates and correction for realignments and mapping quality score recalibrations. Haplotype Caller was used for variant calling. The WGS data were deposited in National Genomics Data Center (https://bigd.big.ac.cn/gsa/browse/CRA005128.).

Twenty-one potential off-target sites for the three sgRNAs targeting *Sik3* and *NeuN* genes listed below were identified using Cas-OFFinder (http://www.rgenome.net/cas-offinder/).

*Sik3* sgRNA-1(ACAGACCACTTGGTAGCCCA):

1. AagGACCACTTGtTAGCCCAAGG chr2:87098654

2. ACAaAgCACTTGGTAGCCtAAGG chr4:46756223

3. ACAGACCACgTGtTAGaCCAAGG chr4:57954753

4. gCAGAaCACTGTGGTAGCCCAGGG chr4:126131919

5. AtA-ACCACTTGGTAGgCCAGGGAGG chr17:86795878

6. ACAGACCAaTctGT-GCCCAGGGTGG chr8:122648253

7. ACAGACCACaTGG-AGCtCAAGaAGG chr15:5662796

*Sik3* sgRNA-2(ACTCACCCATATGTCCACTT):

8. ACTCAgCCATtTGTCCAtTTGGG chr7:123562465

9. ACTCACtCATATGTgCACTgGGG chr2:25603475

10. ACTCACCCtTAgGgCCACTTGGG chr12:17052697

11. ACaCAgCCAgATGTCCACTTGGG chr17:60959507

12. ACTCACaaATATG-CCAgTTTGGTGG chr15:16585273

13. tCTCAgCCATATGTCCACTTC-cAGG chr5:136277718

14. A-TCACCCtTATGTgCtCTTTGGTGG chr7:66324551

*Sik3* sgRNA-3(GTTCAAACAGATCGTCACAG):

15. GTTCAAACAGATCGaaACgGAGG chr5:52797383

16. GTTCAcACAGtTaGTCACAGTGG chr5:144210416

17. GTTCtAACAaATtGTCACAGGGG chr1:49457652

18. GcTCAAACAtATtGTCACAGGGG chr4:17192911

19. GTCTCAAACAcATCGgCACAGGGG chr8:4772824

20. GTCTCAAACAcATCGaCACAGGGGchr1:163033701

21. GaTCAAACAGATaGTACAGAGG chr15:77931180

*NeuN* sgRNA-1(TCGGGGTCCCTGAACCGGAA):

1. TCtGGaTCCCgGAACCGGAAAGG chr16:7277126

2. TCGGGGTCCCTGAACCactAAGG chr7:30375662

3. TaGGGGTCCCTGAAaCaGAATGG chr7:45640152

4. TgGGGtTCCCTGAACCccAAAGG chrX:11971373

5. TCTGGGGTCCCTGAACCtGcAAGG chr17:48361361

6. TCGGGGTCCCaGCAACCaGAAGGG chr5:99874083

7. TCGGGGaCCCgGAACCTGGAAGGG chr5:109528520

*NeuN* sgRNA-2(GCTCAGATGCTGACCGAGCC):

8. GCTCAGATGCTGACaGAcCtGGG chr16:25376832

9. GCTgAGATGCaGACtGAGCCTGG chr16:30614345

10. GCTCAGcTGCTGgCCcAGCCTGG chr15:82930003

11. GCTtAGATGCTGAtgGAGCCTGG chr7:101383685

12. GCTCAGATGCTGgCCTGtGCCAGG chr9:120120623

13. GCTCAGATGtTGtCCAGAGCCAGG chr5:66096471

14. GCTCAGGcTGCTGACaGAGCtGGG chrX:11232414

*NeuN* sgRNA-3(GCTGAATGGGACGATCGTAG):

15. GCTGttTGGGAgGATCGTAGAGG chr13:48330995

16. GCTGtATGGtAgGATCGTAGGGG chr4:22110214

17. GCTGAATGGGAgGATCaTcaGGG chrX:98480354

18. GCTGAATGGagCGATgGgAGAGG chr15:10917958

19. GCTGGAATGtGAgGATaGTAGTGG chrX:164503183

20. GCTGAATGAGGAaGATaGTgGTGG chr5:35422881

21. GCTGgATGGGACGATgGTACtTGG chr8:28174078

#### EEG/EMG surgery

All EEG/EMG surgeries were performed by experienced technicians. Eleven- to 13-week-old male or female mice (all experiments were conducted with male mice if not indicated otherwise) were anesthetized by isoflurane (4% for induction, 2% for maintenance), and surgical tools were sterilized by ethanol just before use. After confirming the mice lack of pain, the head region was shaved, cleaned with ethanol, and the skull was exposed. The exposed skull was cleaned by cotton swabs to improve binding of skull and dental cement. Handheld electrical drill was moved to the λ point and set the coordinate as (0, 0, 0). Then four holes were drilled by the electrical drill in the skull. The coordinate of the holes were as follows: (−1.27, 0, 0), (−1.27, 5.03, 0), (1.27, 5.03, 0), and (1.27, 0, 0). Then the EEG electrode pins were implanted to the dura under stereotaxic control and the EMG wires were inserted into the neck muscle and then stick to the skull with dental cement. After surgery, the mice were housed individually to recover for 1 week. Then retro-orbital injection of AAV-PHP.eB was performed. After allowing time for virus expression (2 weeks for ABC-expression and 3 weeks for ABC-KO), the mice were tethered to a counterbalanced arm (Instech Laboratories) that allowed free movement and exerted minimal weight for 1 week before EEG/EMG recording.

#### EEG/EMG recording and data analysis

Three days of baseline EEG/EMG recording were conducted after mice were acclimated for 1 week in recording condition. For the ABC-*Ox1r/Ox2r^DKO^* mice, an additional 1 week EEG/EMG recording was conducted with feeding chocolates. EEG/EMG raw data were analyzed using a custom semiautomated C++-based sleep/wake staging software followed by manual inspection and correction. Sleep/wake states were classified into NREMS (high amplitude, 1-4 Hz δ frequency EEG and low EMG tonus), REMS (high amplitude, 6-9 Hz theta frequency EEG and EMG muscle atonia), and wake (low amplitude, fast EEG and high amplitude, variable EMG signal). The total time of NREMS, REMS, and wake of each mouse was averaged for the 3 d of EEG/EMG recording. For the hourly plot of relative NREMS δ power density, we calculated the sum of 1-4 Hz EEG signals and divided by the sum of 1-30 Hz EEG signals, and averaged this ratio (relative NREMS δ power density) for all NREMS episodes for every hour over a 3 d baseline recording period. For EEG power spectra analysis, the EEG power of each frequency bins was calculated as the percentage of total EEG power (1-30 Hz) during NREMS, REMS, or wake states.

#### Brain lysate preparation and immunoblotting

Mouse brains were quickly dissected and flash frozen in liquid nitrogen. Brain tissues were homogenized using mortar/pestle with liquid nitrogen and then lysed in ice-cold RIPA buffer (50 mm Tris-HCl, pH 7.4, 150 mm NaCl, 1% Triton X-100, 0.1% SDS) (Beyotime, P0013B) freshly supplemented with protease inhibitors (Roche, 11836145001) and phosphatase inhibitors (Roche, 4906837001) for 30 min and centrifuged at 20,000 × *g* for 15 min at 4°C. The supernatant was transferred to a new tube and boiled at 95°C for 10 min with SDS-loading buffer. Western blotting was performed according to standard protocols. Rabbit polyclonal anti-SIK3 antibodies were generated using Abcam custom antibody production service. The following antibodies were purchased from commercial sources: anti-CREB (CST, 9197S), anti-CRTC1 (CST, C71D11), anti-CDK5 (Santa Cruz Biotechnology, sc-6247), anti-NeuN (Milipore, ABN78), anti-β-actin (Beyotime, AF003), anti-tubulin J (CST, 5568), anti-GFP (Beyotime, AG279), anti-Cas9 (Abcam, ab204448), anti-HA (Sigma, H6533), anti-GAPDH (EarthOx, E021010-01), HRP-labeled goat anti-rabbit IgG(H + L) (Beyotime, A0208), HRP-labeled goat anti-mouse IgG(H + L) (Beyotime, A0216), AP-labeled goat anti-rabbit IgG (H + L) (Beyotime, A0239), and AP-labeled goat anti-mouse IgG(H + L) (Beyotime, A0258). BeyoECL Plus (Beyotime, P0018M) was used for the coloration of HRP-based secondary antibodies. BCIP/NBT Alkaline Phosphatase Color Development Kit (Beyotime, C3206) was used for the coloration of alkaline phosphatase-based secondary antibodies.

#### Immunohistochemistry

Mice were deeply anesthetized and perfused transcardially with 0.9% normal saline followed by 4% PFA in PBS. Brains were postfixed in 4% PFA in PBS at room temperature for at least 4 h followed by incubation in 30% sucrose in PBS at room temperature for 24 h. The cryo-protected brains were sectioned at 40 μm on a cryostat microtome (Leica). After washing in PBST (0.3% Triton X-100 in PBS) for 5 min 3 times, brain sections were incubated in blocking solution (3% BSA, 0.3% Triton X-100 in PBS) at room temperature for 1 h. Then brain sections were incubated with the primary antibodies overnight at 4°C and immunofluorescence-tagged secondary antibodies at room temperature for 2 h. After staining, the brain sections were mounted on adhesion microscope slides (Genview) and encapsulated in sealed tablets containing 3 µg/ml DAPI (Solarbio, C0060).The following antibodies were used: anti-HA (1:500, 11867423001, Roche), anti-NeuN (1:500, ABN78, Milipore), anti-GFP (1:2000, ab13970, Abcam), AlexaFluor-555 conjugated goat-anti-rat (1:500, 4417, CST), AlexaFluor-488 conjugated goat-anti-rabbit (1:500, 111-545-003, Jackson ImmunoResearch Laboratories), and AlexaFluor-488 conjugated goat-anti-chicken (1:500, ab150169, Abcam).

#### RNA extraction and RT-PCR

Mouse brain tissues were quickly dissected and frozen in liquid nitrogen. Previously sterilized mortar and pestle were used to grind brain tissues in liquid nitrogen to fine powder. The brain homogenates were quickly transferred to a new cold 1.5 ml centrifuge tube, and 1 ml TRIzol (Invitrogen, 15596026) was added followed by RNA extraction according to manufacturer's instructions. RNA concentration was quantified by NanoDrop One (Thermo Scientific, ND-ONE-W). Typically, 2 µg RNA sample was used for reverse transcription by QuantScript RT Kit (TIANGEN Biotech, KR103); 2 × iTaq Universal SYBR Green Supermix (Bio-Rad, 1725125) was used for real time qPCR based on the CFX96 Touch qPCR Detection System (Bio-Rad, 1855196). Data were analyzed by CFX Manager software (Bio-Rad).

### Quantification and statistical analysis

Statistical analysis of EEG/EMG data were performed using GraphPad Prism 8.0.2. ImageJ software was used to quantify the KO efficiency as measured by Western blotting. Two-tailed *p* value was used for unpaired *t* test, one-way ANOVA for multiple comparisons, and two-way ANOVA for multiple comparisons involving two independent variables. Dunnett's test compares every mean to a control mean. *p* < 0.05 was considered statistically significant.

## Results

### ABC platform for somatic genetics analysis of sleep in adult mice

**Figure 1.**
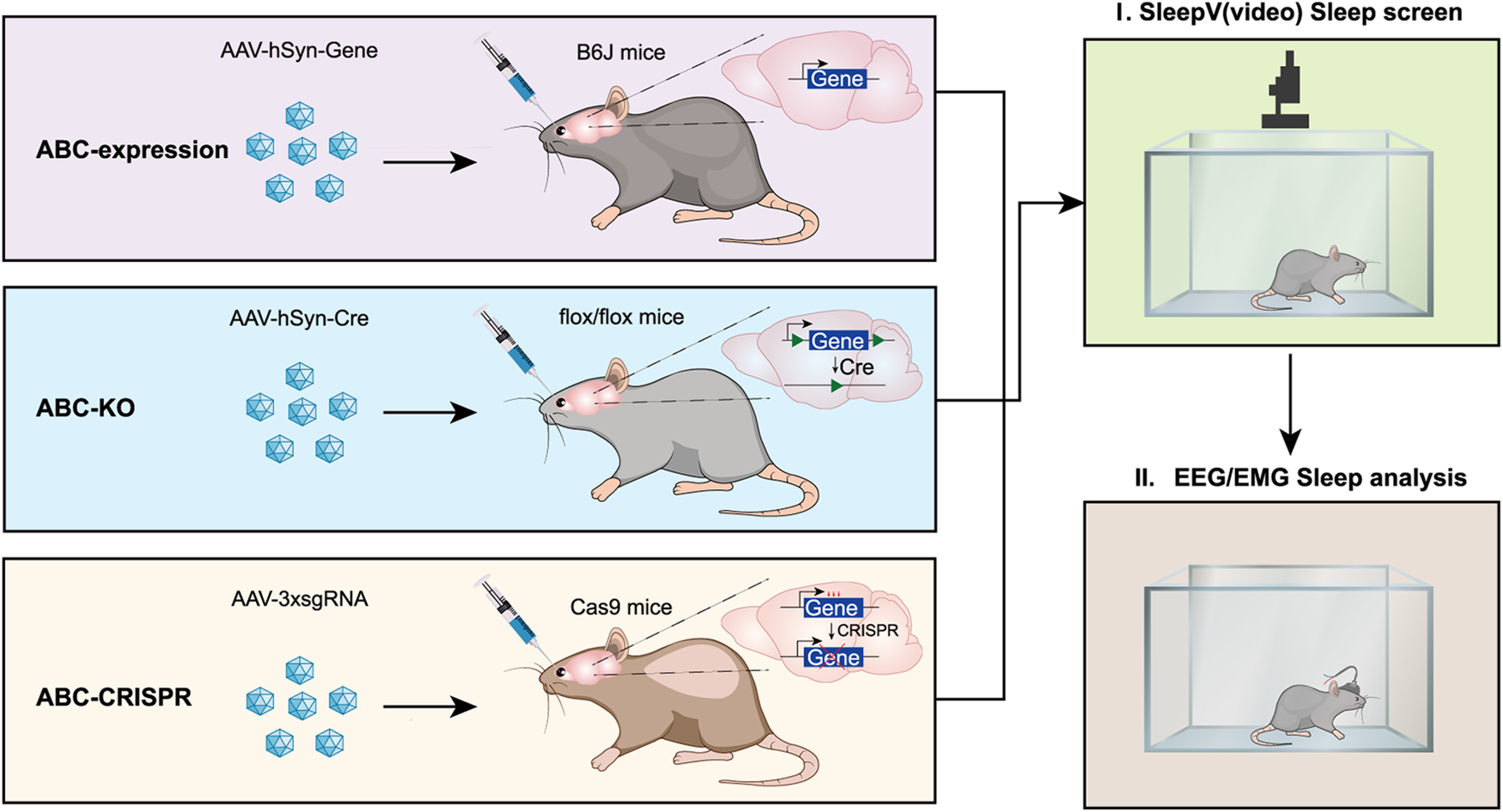
ABC platform for somatic genetics analysis of sleep genes in adult mice. ABC-expression in C57BL/6J adult mice by retro-orbital injection of AAV-PHP.eB to deliver systemic gene expression from the human synapsin (hSyn) promoter in the adult brain neurons. ABC-KO of target gene in conditional flox/flox adult mice by retro-orbital injection of AAV-PHP.eB-expressing Cre recombinase. Multiplex ABC-CRISPR of target genes by retro-orbital injection of AAV-3xsgRNA expressing a set of triple sgRNAs targeting different exons of the same gene. The ABC-expression/KO/CRISPR mice are subjected to high-throughput sleep analysis by SleepV (video) system followed (or directly) by EEG/EMG recording.

Sleep and wakefulness are two alternate physiological states of the brain, which globally impact the molecular, synaptic, and cellular activities across the whole brain (Cirelli and Tononi, 1998; Elliott et al., 2014; Tononi and Cirelli, 2014; de Vivo et al., 2017; Diering et al., 2017; Z. Wang et al., 2018). It is likely that homeostatic sleep regulation involves the majority of neurons and possibly astrocytes across the adult mouse brain (Tononi and Cirelli, 2014; Z. Wang et al., 2018). It has been reported that retro-orbital injection of 10^11^ vector genomes (vg) of single-stranded AAV-PHP.eB systemically transduced ∼40%-80% of adult brain neurons and astrocytes (Chan et al., 2017). Therefore, we hypothesized that AAV-PHP.eB-mediated ABC-expression or KO of sleep regulatory genes should theoretically result in significant sleep phenotypes (Fig. 1).

**Figure 2.**
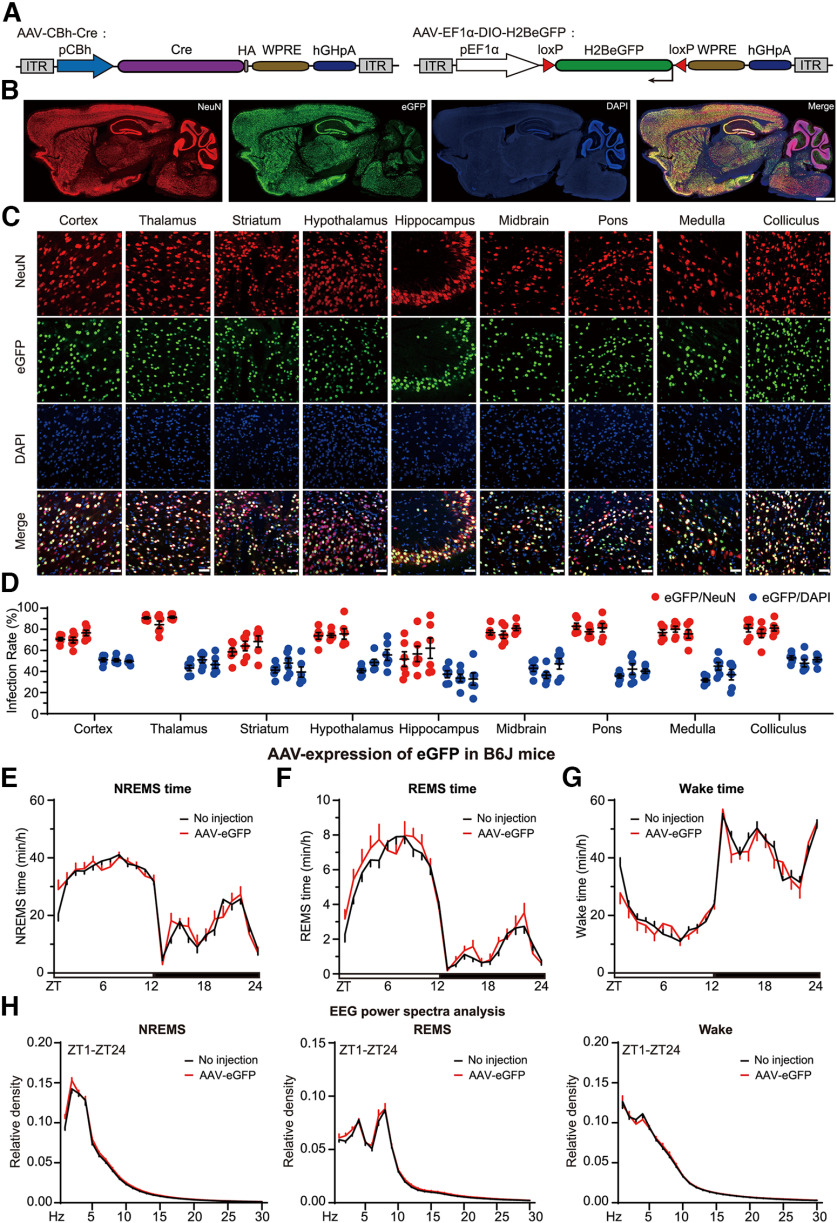
Development of ABC platform for somatic genetics analysis of sleep genes. ***A***, Schematic of the AAV-CBh:Cre and AAV-pEF1α:DIO-H2B-eGFP constructs. ***B***, Representative images showing coimmunostaining of eGFP and NeuN in the sagittal brain sections of AAV-CBh-Cre and AAV-EF1α-DIO-eGFP coinjected mice. ***C***, Representative images of NeuN^+^ (red) neurons or DAPI^+^ (blue) cells that also express eGFP in nine different brain regions. ***D***, Quantification of the viral transduction rates, which is calculated by the percentage of NeuN^+^ neurons (red) and DAPI^+^ (blue) cells that express eGFP, in nine brain regions shown in ***C***. ***E–H***, Hourly plots of NREMS (***E***), REMS (***F***), or Wake (***G***) time and EEG power spectra analysis of NREMS, REMS, and Wake states (***H***) of no virus (*n* = 19) or AAV-hSyn-eGFP (*n* = 9) injected mice. Data are mean ± SEM. ***E–H***, Two-way ANOVA with Dunn's multiple comparisons test.

To verify the efficiency of this AAV delivery system, we performed retro-orbital injection of 12-week-old C57BL/6J mice with dual AAV-PHP.eB (10^12^ vg/mice), AAV-CBh-Cre, and AAV-EF1α-DIO-H2B-eGFP (Fig. 2*A*). Thus, only brain cells cotransduced with both AAVs could exhibit Cre-dependent expression of histone H2B-GFP fusion proteins. As shown by coimmunostaining of GFP and NeuN, a pan-neuronal marker protein (Fig. 2*B*,*C*), these two viruses efficiently cotransduced the majority of the adult brain neurons 2 weeks after AAV administration. Quantification of double positive cells showed a high percentage of GFP-expressing neurons across nine different brain regions, ranging from averaging 56.6% in the hippocampus to 88.7% in the thalamus (Fig. 2*D*). More importantly, ABC-expression of the GFP reporter did not affect the sleep/wake architecture in the AAV-PHP.eB-injected mice relative to no virus injected control mice (Fig. 2*E-H*), suggesting that it is feasible to use the ABC-expression/KO platform for rapid and efficient somatic genetics analysis of sleep regulatory genes.

### A highly accurate SleepV (video) system expedites mouse sleep screening

The EEG/EMG recording is the “gold standard” method for sleep/wake analysis in mammals (Lo et al., 2004; Weiergraber et al., 2005). Based on the different patterns of electrical signals, each short (4-20 s) epoch of EEG/EMG data are classified into one of three states: wakefulness (Wake), REMS, or NREMS. NREMS, which occupies ∼90% of total sleep time in mice, is characterized by high percentage of the δ (1-4 Hz) power of EEG spectrum. The δ power of NREMS measures sleep quality or depth, which is often regarded as a good index of homeostatic sleep need (Franken et al., 2001).

However, this EEG/EMG method is not optimal for high-throughput mouse sleep screening because it requires labor-intensive and invasive surgery to implant electrodes into the skull and muscle and extensive recovery time from surgery before EEG/EMG recording. Moreover, the semiautomated sleep staging software only has ∼90% accuracy and requires intensive efforts to manually correct the annotation of several days of EEG/EMG data per mouse. On the other hand, a number of noninvasive sleep monitoring systems have been developed and used for mouse sleep analysis, including the infrared video recording system (Pack et al., 2007; Fisher et al., 2012; Banks et al., 2020), the piezoelectric system tracking mouse movement (Flores et al., 2007; Yaghouby et al., 2016; Joshi et al., 2019) and the plethysmography system monitoring the respiration of mouse (Sunagawa et al., 2016).

**Figure 3.**
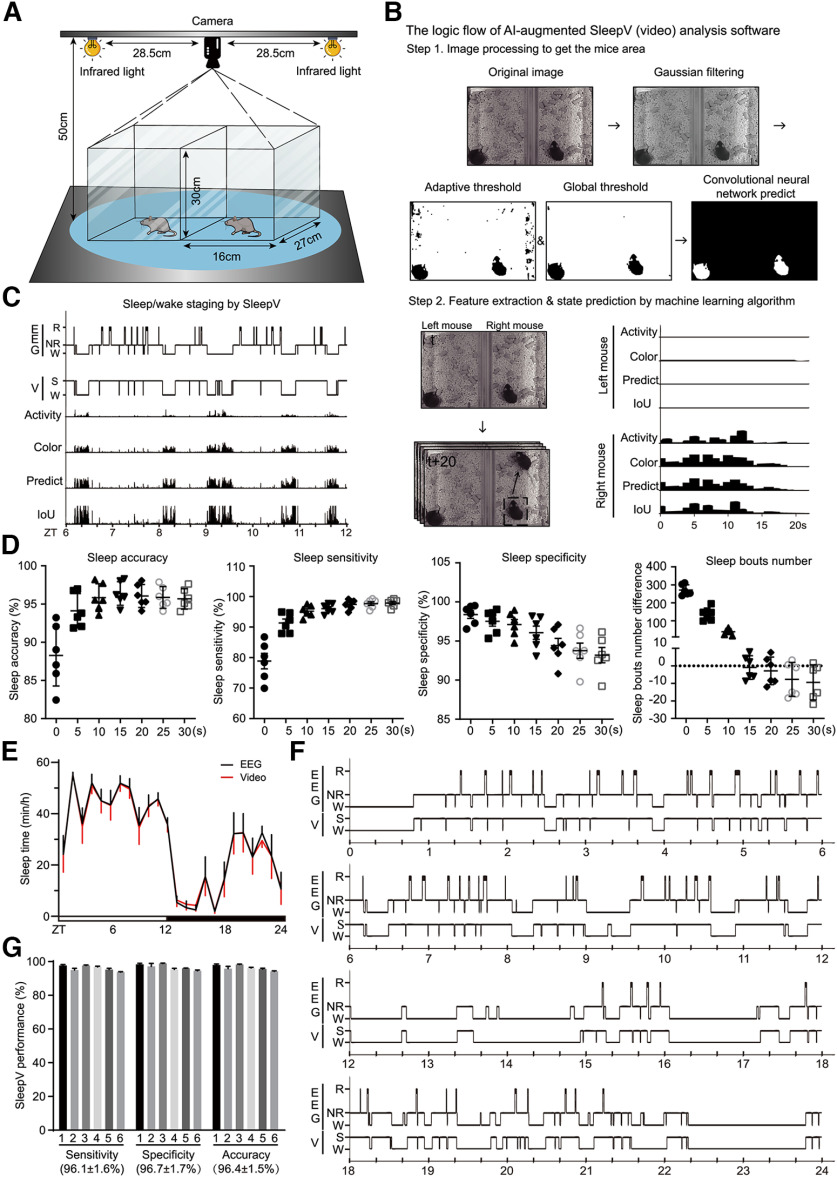
Development of a SleepV(video) system. ***A***, Schematic diagram of detailed setup information of SleepV. ***B***, The logic flow of sleep/wake staging of video recording by SleepV software. ***C***, Epoch-by-epoch comparison of sleep/wake staging and parameter changes of the same mouse by simultaneous SleepV and EEG/EMG recording and analysis. ***D***, Graphs represent the accuracy, sensitivity, specificity, and sleep bout number of sleep/wake staging by SleepV after filtering 0, 5, 10, 15, 20, 25, or 30 s of small movements during sleep. ***E***, Hourly plot of sleep time of 6 C57BL/6J mice by simultaneous SleepV(video) and EEG/EMG analysis. ***F***, Epoch-by-epoch comparison of sleep/wake staging of the same mouse by simultaneous SleepV and EEG/EMG analysis. ***G***, Quantitative analysis of the sensitivity, specificity, and accuracy of sleep/wake staging of 6 mice by SleepV (total 77,760 epochs, 20 s/epoch). Data are mean ± SEM. ***E***, Two-way ANOVA with Dunn's multiple comparisons test. For comparison of sleep/wake staging by EEG/EMG and SleepV analysis, see Movie 1. For filtering of small movement during sleep by SleepV system, see Movie 2.

Here, we developed an artificial intelligence-augmented video-based sleep monitoring system that we named SleepV, which used a novel pattern recognition algorithm for sleep/wake staging based on inactivity/activity of the mouse (Fig. 3*A*). First, the SleepV algorithm uses Gaussian filtering, adaptive and global thresholding to extract from every image frame (25 frames/s) to the suspected ROIs, which are then judged to be a mouse or not by a pretrained deep neural network. Second, to determine whether the mouse is active or not, the algorithm calculates a high confidence difference score for the mouse between “t” and “t + m” frames by integrating the network prediction score (predict), the mouse mask area IoU, the center of mass of the mask (activity), and the gray information (color) within the detected mask (Fig. 3*B*,*C*). Finally, SleepV defines the sleep state as ≥40 s of continuous immobility similar to previously reported video-based sleep monitoring systems (Pack et al., 2007; Fisher et al., 2012; Banks et al., 2020).

We performed simultaneous infrared video recording and EEG/EMG recording on six C57BL/6J adult mice to examine the accuracy of sleep/wake staging by fully automated SleepV analysis (Movie 1). Compared with semiautomated EEG/EMG analysis with manual corrections, SleepV showed suboptimal accuracy (average 88.2%) by overestimating the number of sleep bouts owing to misjudging small mouse movements during sleep as waking (Fig. 3*D*). To improve the performance of SleepV, we systematically tested a series of thresholds of 0, 5, 10, 15, 20, 25, and 30 s to filter subtle mouse movements during sleep, and compared the number of sleep bouts as well as the accuracy, sensitivity, and specificity of sleep/wake staging (Fig. 3*D*; Movie 2). Notably, we found that the best performance of SleepV was achieved with a 15 s threshold, that is, by annotating ≤15 s of activity between two sleep bouts as sleep. With this improvement, the accuracy of sleep/wake staging by SleepV was now comparable with that of semiautomatic EEG/EMG analysis with human corrections (Fig. 3*E*). Although SleepV could not distinguish between NREMS and REMS, there was a remarkable 95%-99% epoch-by-epoch agreement between the two methods, with ∼96% in sensitivity, specificity, and accuracy of sleep/wake staging by SleepV (Fig. 3*F*,*G*). The mean accuracy (96.4%) of SleepV is significantly higher than that (92%-94%) of previous reported video-based sleep monitoring systems (Pack et al., 2007; Fisher et al., 2012; Banks et al., 2020).

Movie 1. SleepV can easily detect sleep/wake state transition. Shown are EEG/EMG characterized behavior states, SleepV characterized behavior states, and real-time color&activity changes. NREM (N)-Wake (W)-NREM transitions were observed on the left mouse. REM (R)-Wake (W) transition was observed on the right mouse. The video is shown at 3× the original speed. S, Sleep. 10.1523/JNEUROSCI.0089-22.2022.video.1

Movie 2. SleepV can filter small movement during sleep. Shown are EEG/EMG characterized behavior state, SleepV characterized behavior state, and real-time color&activity changes. Both the left and right mice showed small movement during NREM sleep, which could be filtered by SleepV. The video is shown at 3× the original speed. 10.1523/JNEUROSCI.0089-22.2022.video.2

**Figure 4.**
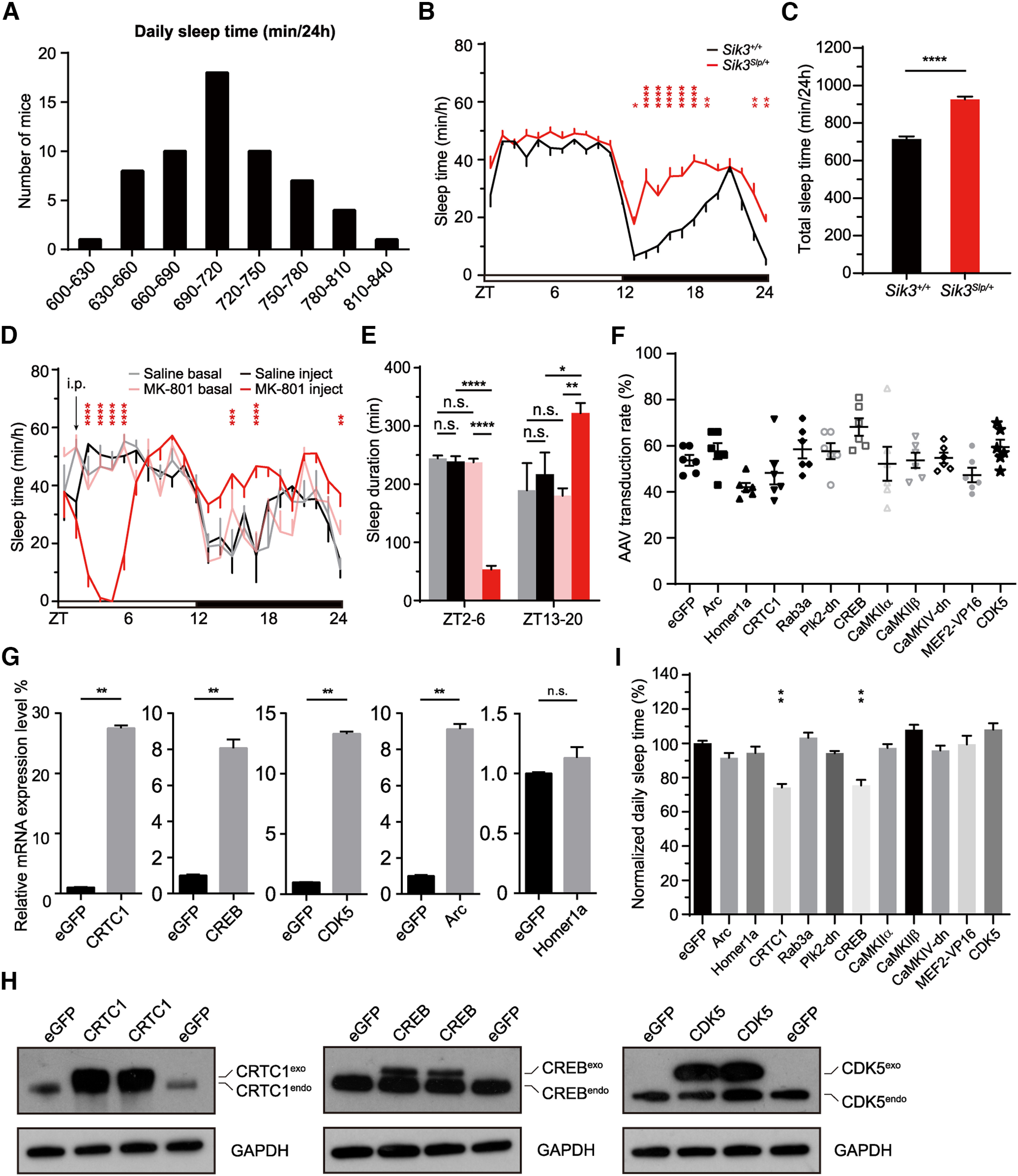
A high-throughput ABC sleep screening platform using SleepV system. ***A***, Distribution of daily sleep time in 59 C57BL/6J mice analyzed by SleepV. ***B***, Hourly plot of sleep time of *Sik3*^+/+^ (*n* = 11) and *Sik3*^*Slp*/+^ (*n* = 11) mice analyzed by SleepV. ***C***, Quantification of daily sleep time in *Sik3*^+/+^ (*n* = 11) and *Sik3*^*Slp*/+^ (*n* = 11) mice. ***D***, Hourly plot of sleep time of baseline condition or after intraperitoneal injection of saline (black, *n* = 4) or 2 mg/kg MK-801 (red, *n* = 4) at ZT2 in C57BL/6J mice. ***E***, Quantification of sleep time during ZT2-6 and ZT13-20 after saline or MK-801 injection. ***F***, Graph represents viral transduction rates of cortical neurons in AAV-hSyn-GeneX-injected mice. ***G***, qRT-PCR analysis of the *Crtc1*, *Creb*, *Cdk5*, *Arc*, or *Homer1a* mRNA level in the ABC mouse brains. ***H***, Immunoblotting of CRTC1, CREB, and CDK5 proteins in the ABC mouse brains. ***I***, Graph represents daily sleep time of ABC-GeneX mice (*n* ≥ 5) normalized to ABC-eGFP mice. Data are mean ± SEM. ***B***, ***D***, ***E***, Two-way ANOVA with Dunn's multiple comparisons test. ***C***, ***G***, Unpaired *t* test. ***I***, One-way ANOVA with Dunn's multiple comparisons test. *p < 0.05; **p < 0.01; ****p < 0.0001.

Next, we used SleepV to record the sleep/wake cycles of 59 C57BL/6J adult mice for 3 consecutive days. The distribution of daily sleep time measured by SleepV in these mice was comparable with that of EEG/EMG analysis in previous studies (Fig. 4*A*) (Funato et al., 2016; Z. Wang et al., 2018). Moreover, SleepV could easily distinguish the hypersomnia phenotype of *Sleepy* (*Sik3*^*Slp*/+^) mice, which showed on average ∼210 min increase in daily sleep time relative to WT littermates (Fig. 4*B*,*C*). To test whether SleepV could detect dynamic changes in sleep/wake behavior, we intraperitoneally injected C57BL/6J mice with 2 mg/kg MK-801, a specific inhibitor of NMDAR, at zeitgeber time 2 (ZT2) (Sunagawa et al., 2016; Tatsuki et al., 2016; Z. Wang et al., 2018). Compared with the baseline and saline injection controls, MK-801 treatment rapidly decreased sleep time during ZT2-6, which was followed by rebound sleep during ZT13-20 (Fig. 4*D*,*E*). Collectively, these results demonstrate that SleepV is a highly accurate, noninvasive, and fully automatic sleep monitoring system that is suitable for high-throughput mouse sleep screening.

### A pilot ABC-expression sleep screen of synaptic plasticity regulators

Accumulating studies suggest a close link between synaptic plasticity and sleep need regulation (Huber et al., 2004, 2006; Ganguly-Fitzgerald et al., 2006; Donlea et al., 2009). For example, fruit flies raised in socially enriched environment sleep for significantly longer time than those raised in isolation (Ganguly-Fitzgerald et al., 2006; Donlea et al., 2009). Learning experience also increase sleep need in both flies and mammals, manifested as increased sleep time and/or NREMS δ power (Huber et al., 2004, 2006; Ganguly-Fitzgerald et al., 2006; Donlea et al., 2009). Quantitative phosphoproteomic analyses of *Sleepy* and sleep-deprived mouse brains have identified 80 mostly synaptic sleep need index phosphoproteins, including many regulators of synaptic plasticity (Z. Wang et al., 2018).

To test our hypothesis that changing synaptic plasticity could lead to changes in sleep need, we used SleepV system to conduct a pilot ABC-expression sleep screen of 11 known regulators of synaptic plasticity (Fig. 4*F*). These included the immediate early gene products Arc and Homer1a (Plath et al., 2006; Rial Verde et al., 2006; Shepherd et al., 2006; Hu et al., 2010; Diering et al., 2017), cyclin-dependent kinase 5 (CDK5) (Bibb, 2003), Rab3a (Kapfhamer et al., 2002), dominant negative form of polo-like kinase 2 (Plk2-dn) (Seeburg et al., 2008), CaMKIIα/β and CaMKIV (Ibata et al., 2008; Lisman et al., 2012), activity-dependent transcriptional factors cyclic AMP-response element binding protein (CREB) and CREB regulated transcriptional coactivator 1 (CRTC1), and constitutively active MEF2^VP16^, a fusion protein between the DNA binding domain of MEF2 and VP16 transactivation domain (Flavell et al., 2006; Benito and Barco, 2010; Ch'ng et al., 2012; Kandel, 2012; Nonaka et al., 2014). As shown by coimmunostaining of HA-tag and NeuN, intravenous administration of AAV-PHP.eB consistently resulted in systemic expression of target genes in 40%-80% of neurons across the adult mouse brains (Fig. 4*F–H*). While ABC-expression of most genes had little effect on the sleep/wake cycle, this pilot screen identified two potential hits, CREB and CRTC1, which significantly reduced daily sleep amount (Fig. 4*I*).

CREB is an activity-dependent transcriptional activator that binds as a dimer to the cAMP response elements (CRE), which contain a palindromic (TGACGTCA) or half-site (TGACG or CGTCA) sequence, in the promoter or enhancer regions of target genes (Comb et al., 1986; Montminy et al., 1986; Short et al., 1986). The *Creb1* gene encodes multiple CREB isoforms by alternative splicing, of which the α and Δ isoforms, but not the β isoform, show high affinity for CRE sites (Ruppert et al., 1992). Phosphorylation of CREB_α_ at serine 133 (or CREB_Δ_ at serine 119) by cAMP-dependent protein kinase promotes the recruitment of coactivators, including histone acetyl transferases CBP/p300, and transcriptional activation of certain target genes (Gonzalez and Montminy, 1989; Shaywitz and Greenberg, 1999; Chrivia et al., 1993). Alternatively, CREB functions in tandem with CRTCs, also known as transducers of regulated CREB activity coactivators, to activate the transcription of a specific subset of target genes (Conkright et al., 2003; Iourgenko et al., 2003).

### ABC-expression of CREB and/or CRTC1 reduces NREMS amount and δ power

**Figure 5.**
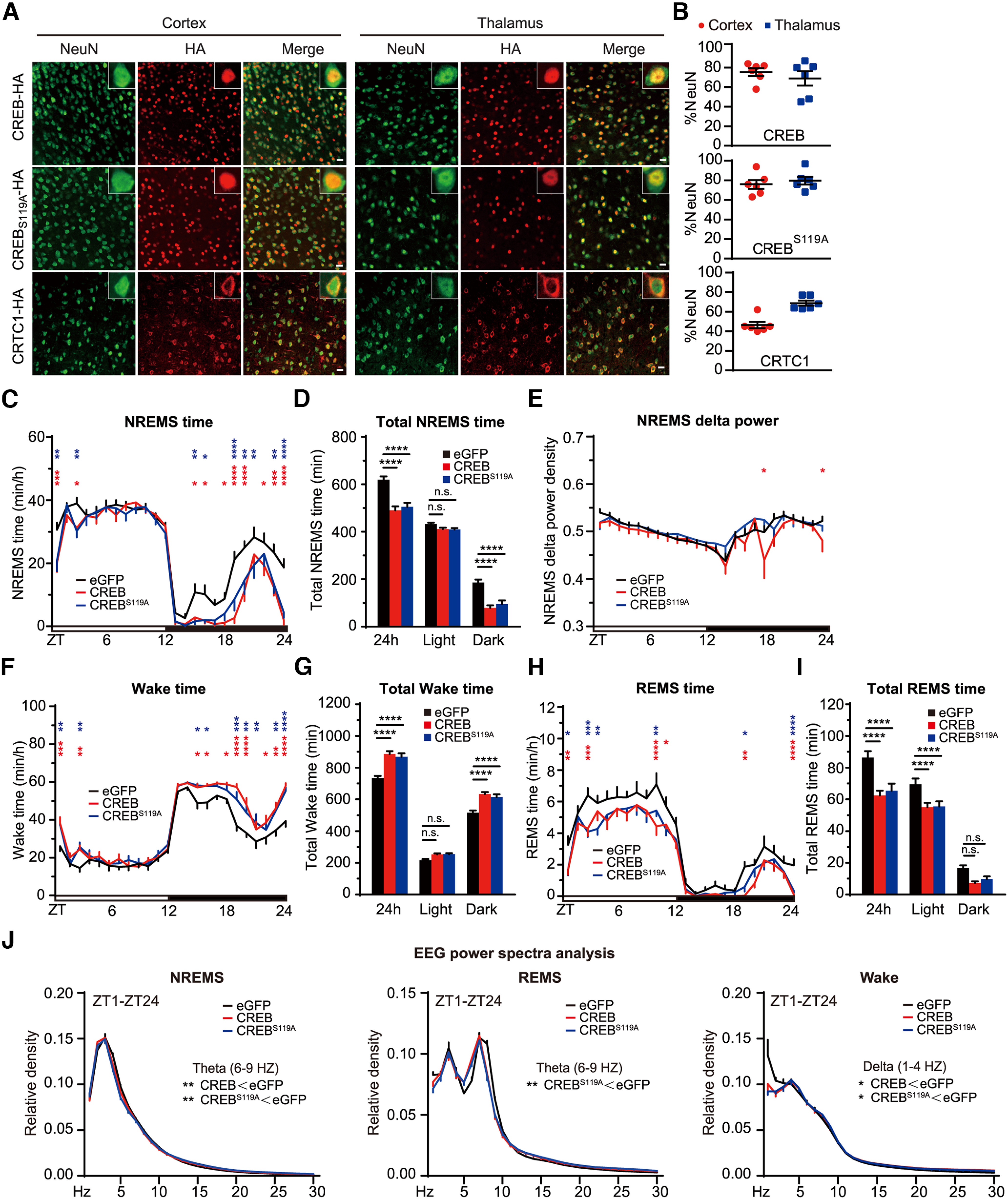
ABC-expression of CREB reduces NREMS amount and δ power. ***A***, Coimmunostaining of HA^+^ (red) and NeuN^+^ (green) neurons in the cortex and thalamus of AAV-hSyn-CREB (ABC-CREB), AAV-hSyn-CREB^S119A^ (ABC-CREB^S119A^), and AAV-hSyn-CRTC1 (ABC-CRTC1)-injected mice. ***B***, Quantification of the viral transduction rates, which is calculated by the percentage of NeuN^+^ neurons that express HA-tagged proteins, in the cortical and thalamic neurons of ABC-CREB, ABC-CREB^S119A^, and ABC-CRTC1 mice. ***C–E***, Hourly plot of NREMS time (***C***), quantification of total NREMS time (***D***), and hourly plot of NREMS δ power (***E***) in the ABC-eGFP (*n* = 11), ABC-CREB (*n* = 12), and ABC-CREB^S119A^ (*n* = 11) mice. Shown above are the statistical analysis for comparison between ABC-CREB (red*) or ABC-CREB^S119A^ (blue*) mice and control ABC-eGFP mice. ***F–J***, Hourly plots of Wake (***F***) or REMS (***H***) time, quantification of total Wake (***G***) or REMS (***I***) time, and EEG power spectra analysis of NREMS, REMS, and Wake states (***J***) in the ABC-eGFP (*n* = 11), ABC-CREB_Δ_ (*n* = 12), and ABC-CREB_Δ_^S119A^ (*n* = 11) mice. Shown above is statistical analysis for comparison between ABC-CREB (red*) or ABC-CREB^S119A^ (blue*) mice and control ABC-eGFP mice. Data are mean ± SEM. ***C–J***, Two-way ANOVA with Dunn's multiple comparisons test. *p < 0.05; **p < 0.01; ***p < 0.001; ****p < 0.0001.

We performed EEG/EMG recording to further characterize the sleep phenotypes caused by ABC-expression of CREB_Δ_. It has been shown that serine 133 to alanine (S133A) phosphor-mutation of CREB_α_ prevents transcriptional activation of specific target genes (Gonzalez and Montminy, 1989; Chrivia et al., 1993). Thus, we compared the sleep phenotypes as a result of ABC-expression of WT or S119A phosphor-mutant CREB_Δ_ in C57BL/6J adult mice. Coimmunostaining revealed that intravenous administration of AAV-hSyn-CREB_Δ_ or AAV-hSyn-CREB_Δ_^S119A^ resulted in efficient transduction of the majority of cortical and thalamic neurons (Fig. 5*A*,*B*). Both ABC-CREB_Δ_ and ABC-CREB_Δ_^S119A^ mice, relative to ABC-eGFP mice, exhibited on average ∼120 min decrease in daily NREMS amount, which occurred mostly during the dark phase (Fig. 5*C–J*). These results suggest that ABC-expression of CREB_Δ_ reduces NREMS amount in a manner independent of S119 phosphorylation.

**Figure 6.**
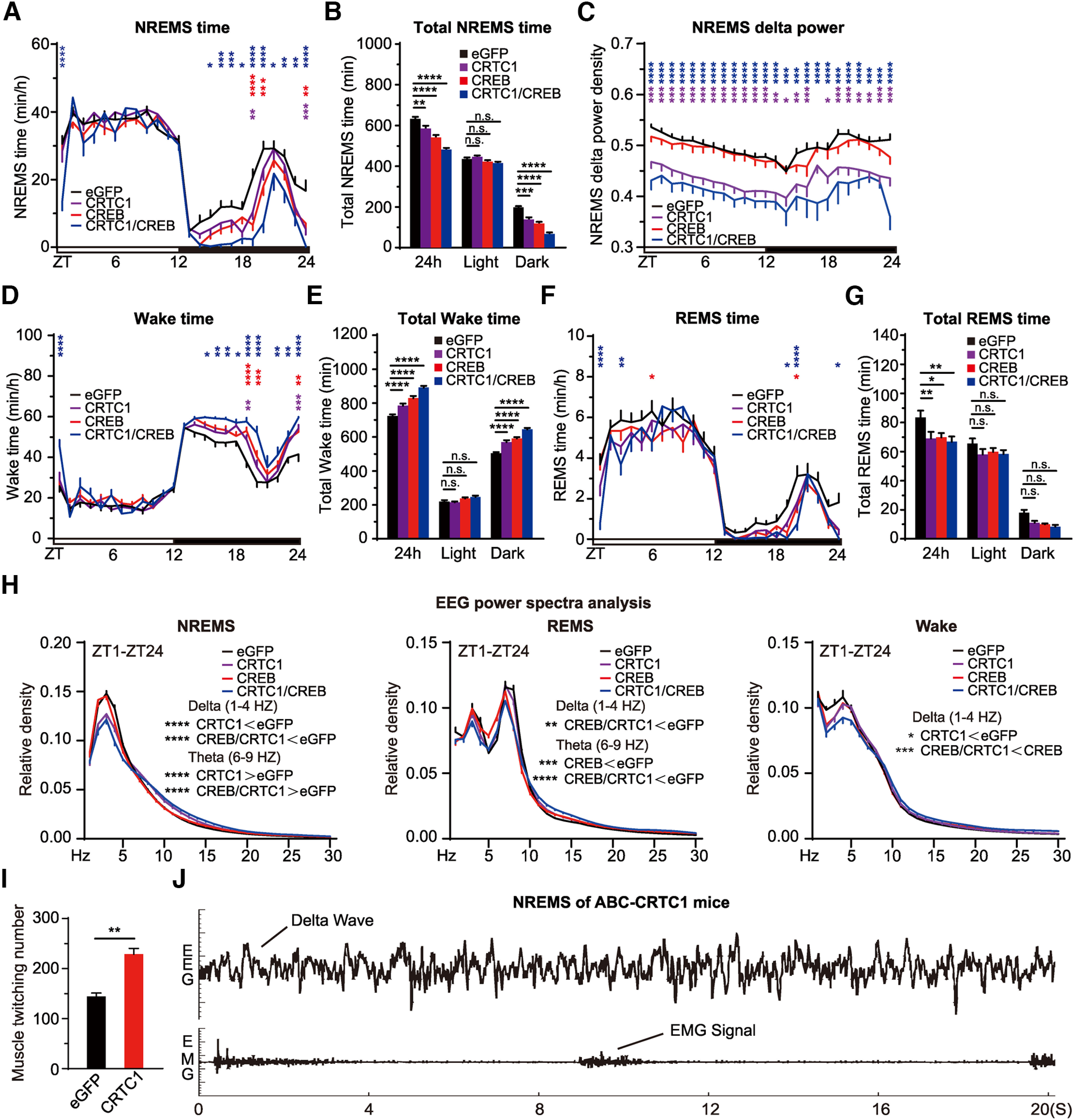
ABC-expression of CREB and/or CRTC1 reduces NREMS amount and δ power. ***A–C***, Hourly plot of NREMS time (***A***), quantification of total NREMS time (***B***), and hourly plot of NREMS δ power (***C***) in the ABC-eGFP (*n* = 12), ABC-CRTC1 (*n* = 15), ABC-CREB (*n* = 15), and ABC-CRTC1/CREB (*n* = 12) mice. Shown above are statistical analysis for comparison between ABC-CRTC1 (purple*), ABC-CREB (red*), or ABC-CRTC1/CREB (blue*) mice and control ABC-eGFP mice. ***D–H***, Hourly plots of Wake (***D***) or REMS (***F***) time, quantification of total Wake (***E***) or REMS (***G***) time, and EEG power spectra analysis of NREMS, REMS, and Wake states (***H***) in ABC-eGFP (*n* = 12), ABC-CRTC1 (*n* = 15), ABC-CREB_Δ_ (*n* = 15), and ABC-CRTC1/CREB_Δ_ (*n* = 12) mice. Shown above is the statistical analysis for comparison between ABC-CRTC1 (purple*), ABC-CREB_Δ_ (red*), or ABC-CRTC1/CREB_Δ_ (blue*) mice and control ABC-eGFP mice. ***I***, Quantification of muscle twitching episodes of ABC-eGFP and ABC-CRTC1 mice during NREMS. ***J***, Representative EEG/EMG hypnogram depicting frequent muscle twitching during NREMS in the ABC-CRTC1 mice. Data are mean ± SEM. ***A–H***, Two-way ANOVA with Dunn's multiple comparisons test. ***I***, Unpaired *t* test. *p < 0.05; **p < 0.01; ***p < 0.001; ****p < 0.0001. For the muscle twitching phenotype of ABC-CRTC1 mice during sleep, see Movie 3.

Next, we asked whether ABC-coexpression of CREB_Δ_ and CRTC1 could result in additive sleep phenotypes compared with ABC-expression of either CREB_Δ_ or CRTC1 alone. In contrast to nuclear localization of CREB_Δ_, CRTC1 was predominantly localized in the cytoplasm (Fig. 5*A*). ABC-CRTC1 mice, relative to ABC-eGFP mice, exhibited on average ∼47 min decrease in daily NREMS time and reduced NREMS δ power during the dark phase (Fig. 6*A–H*). It should be noted that SleepV overestimated the reduction of sleep time in ABC-CRTC1 mice by misjudging excessive muscle twitching during sleep as waking in these mice (Fig. 6*I*,*J*; Movie 3). Importantly, ABC-coexpression of CREB_Δ_ and CRTC1 resulted in additive sleep phenotypes: ∼150 min decrease in daily NREMS time accompanied by even lower NREMS δ power during the dark phase (Fig. 6*A–C*). These results suggest that the function of CREB in sleep regulation is probably facilitated by a CRTC-dependent mechanism. Furthermore, this pilot screen demonstrates the proof of principle that the combination of ABC platform and SleepV system can facilitate high-throughput somatic genetics screening of new sleep regulatory genes in mice.

Movie 3. ABC-CRTC1 mouse shows frequent muscle twitching during sleep. 10.1523/JNEUROSCI.0089-22.2022.video.3

### Inducible ABC-expression of CREB^VP16^ and/or CRTC1^CA^ causes strong sleep phenotypes

**Figure 7.**
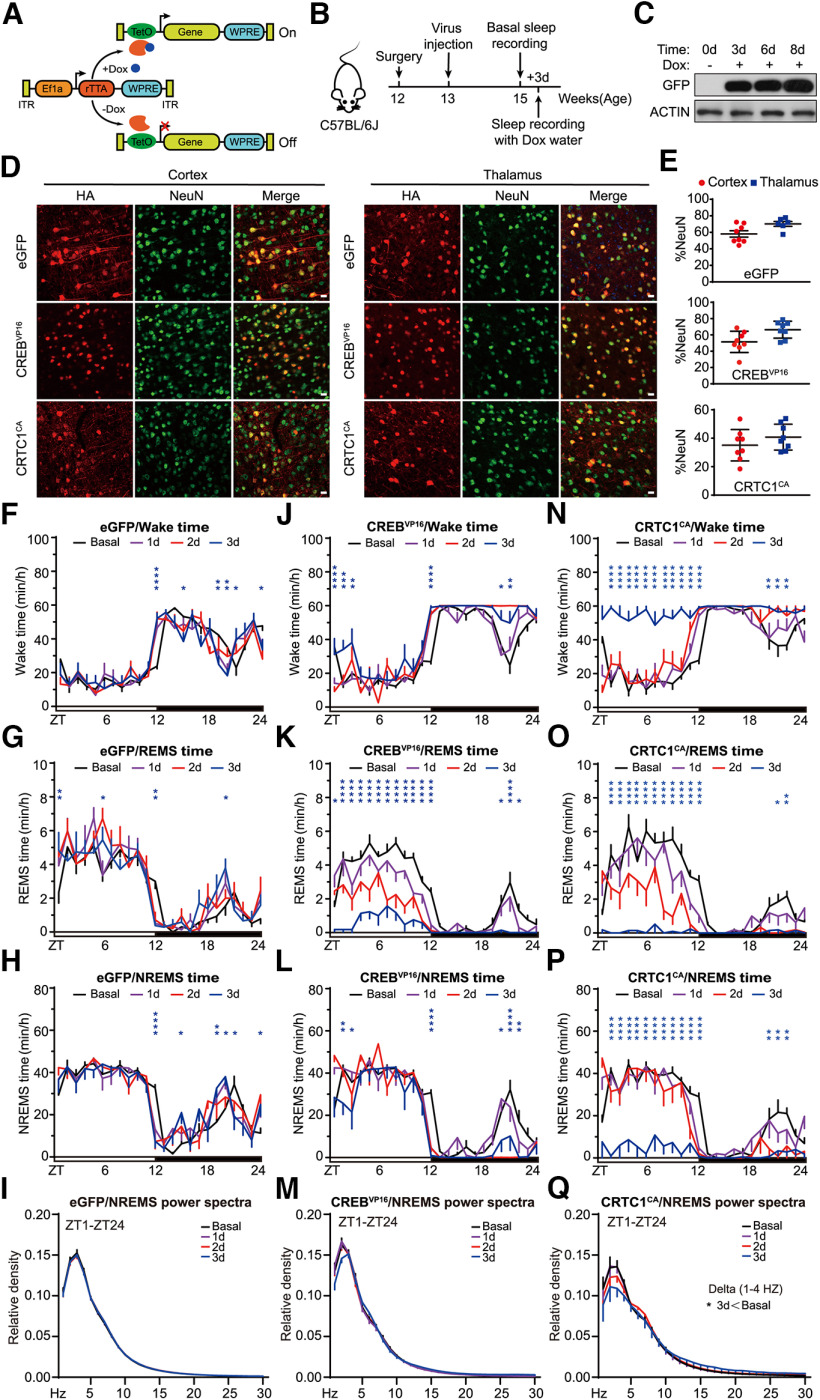
Inducible ABC-expression of CREB^VP16^ or CRTC1^CA^ causes significant sleep phenotypes. ***A***, Schematic of Tet-on inducible (i)ABC-expression system with or without Dox. ***B***, A flowchart of the iABC-expression and EEG/EMG sleep recording experiment. ***C***, Immunoblotting of whole-brain lysates from iABC-eGFP mice before and after Dox treatment with anti-GFP and anti-ACTIN antibodies. ***D***, Coimmunostaining of HA^+^ (red) and NeuN^+^ (green) neurons in the cortex and thalamus of the inducible (i)ABC-eGFP, ABC-CREB^VP16^, and ABC-CRTC1^CA^ mice. ***E***, Quantification of the viral transduction rates, which is calculated by the percentage of NeuN^+^ neurons that express HA-tagged proteins, in the cortical and thalamic neurons showed in ***D***. ***F–I***, Hourly plots of Wake time (***F***), REMS time (***G***), NREMS time (***H***), and EEG power spectra analysis of NREMS (***I***) of the iABC-eGFP mice (*n* = 8). ***J–M***, Hourly plots of Wake time (***J***), REMS time (***K***), NREMS time (***L***), and EEG power spectra analysis of NREMS (***M***) in the iABC-CREB^VP16^ mice (*n* = 7). ***N–Q***, Hourly plots of Wake time (***N***), REMS time (***O***), NREMS time, (***P***) and EEG power spectra analysis of NREMS (***Q***) in the iABC-CRTC1^CA^ mice (*n* = 7). Data are mean ± SEM. ***F–Q***, Two-way ANOVA with Dunn's multiple comparisons test. *p < 0.05; **p < 0.01; ***p < 0.001; ****p < 0.0001.

ABC-expression of constitutively active CREB^VP16^, a fusion protein between the DNA-binding domain of CREB and VP16 transactivation domain (Barco et al., 2002), or constitutively active CRTC1^CA^ containing two (S151A and S245A) phosphor-mutations (Sonntag et al., 2017), resulted in lethality 1 week after AAV injection in C57BL/6J adult mice. To study the immediate sleep phenotypes resulted from ABC-expression of CREB^VP16^ or CRTC1^CA^, we used a Tet-on inducible system to express CREB^VP16^ or CRTC1^CA^ in the adult mouse brains by coinjection of two AAV-PHP.eB viruses expressing rtTA from the EF1α promoter and CREB^VP16^ or CRTC1^CA^ from the TRE promoter, respectively (Fig. 7*A*). There was no difference in the baseline sleep/wake architecture among the inducible (i)ABC-eGFP, iABC-CREB^VP16^ and iABC-CRTC1^CA^ mice when transcription from the TRE promoter was inactive in the absence of Dox (data not shown). On the other hand, the expression of GFP, CREB^VP16^, or CRTC1^CA^ was rapidly induced in the brain cells of these mice within 3 d after drinking Dox-containing water (Fig. 7*B–E*). Interestingly, a slight circadian shift of the sleep/wake cycle was observed at the light/dark transition among all mice possibly because of the effects of Dox (Fig. 7*F–Q*). While ABC-induction of GFP did not affect total sleep/wake time, ABC-induction of CREB^VP16^ or CRTC1^CA^ caused progressive decrease in daily amounts of NREMS and REMS accompanied by corresponding increase in total wake time during 3 d of Dox treatment (Fig. 7*F–Q*). Remarkably, ABC-CRTC1^CA^ mice were almost constantly awake on day 3 of Dox treatment as shown by 91.8% and 97.1% reduction in NREMS and REMS, respectively (Fig. 7*N–Q*). These results suggest that iABC-expression system can be used to study the immediate sleep phenotypes of target genes of which constitutive expression causes rapid lethality in adult mice.

### ABC-KO of *Creb1* by Cre/loxP recombination increases daily NREMS amount

**Figure 8.**
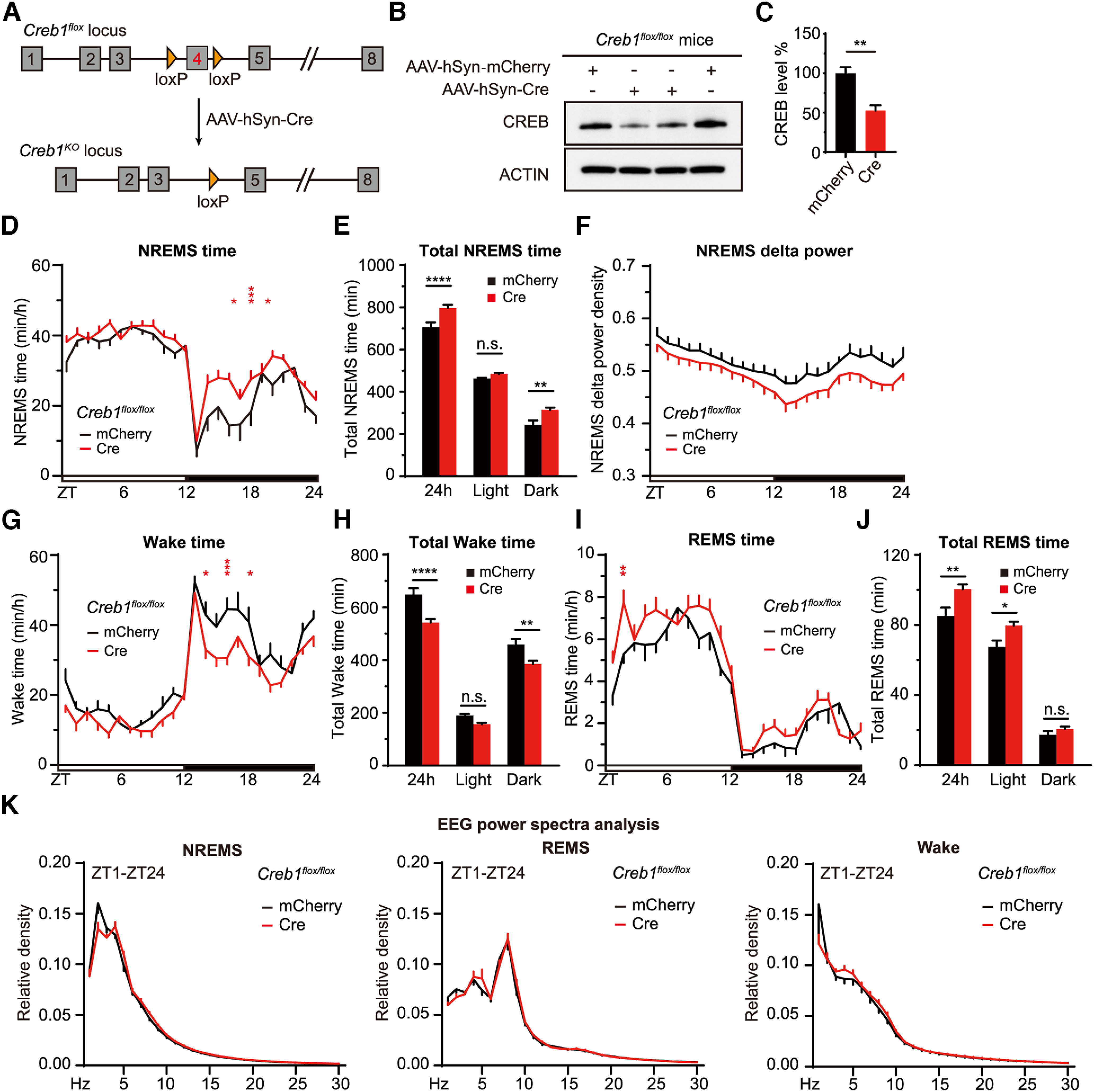
ABC-KO of *Creb1* by Cre-loxP recombination causes hypersomnia. ***A***, Schematic of ABC-KO of *Creb1* by AAV-hSyn-Cre injection of *Creb1^flox/flox^* mice. ***B***, Immunoblotting of brain lysates from AAV-hSyn-mCherry or AAV-hSyn-Cre injected *Creb1^flox/flox^* mice with anti-CREB and anti-ACTIN antibodies. ***C***, Quantification of CREB expression in ***B*** (*n* = 4). ***D–F***, Hourly plot of NREMS time (***D***), quantification of total NREMS time (***E***), and hourly plot of NREMS δ power (***F***) in the AAV-hSyn-mCherry (*n* = 9) or AAV-hSyn-Cre (*n* = 14) injected *Creb1^flox/flox^* mice. ***G–K***, Hourly plots of Wake (***G***) or REMS (***I***) time, quantification of total Wake (***H***) or REMS (***J***) time, and EEG power spectra analysis of NREMS, REMS, and Wake states (***K***) in the AAV-hSyn-mCherry (*n* = 9) or AAV-hSyn-Cre (*n* = 14) injected *Creb1^flox/flox^* mice. Data are mean ± SEM. ***C***, Unpaired *t* test. ***D–K***, Two-way ANOVA with Dunn's multiple comparisons test. *p < 0.05; **p < 0.01; ***p < 0.001; ****p < 0.0001.

*Creb1* is an essential gene of which complete ablation results in perinatal lethality in mice (Bleckmann et al., 2002). A partial *Creb1* KO strain, in which the α and Δ isoforms of CREB are deleted, is homozygous viable and exhibits ∼100 min increase of daily NREMS time (Graves et al., 2003). Moreover, forebrain-specific KO of *Creb1* in the excitatory neurons similarly increases daily NREMS amount (Wimmer et al., 2021). To generate ABC-*Creb1^KO^* mice, we retro-orbitally injected *Creb1^flox/flox^* mice with AAV-PHP.eB-expressing mCherry or Cre recombinase from the pan-neuronal hSyn promoter, respectively. Immunoblotting revealed that the level of CREB expression was reduced by ∼50% in whole-brain lysates of AAV-hSyn-Cre injected mice relative to AAV-hSyn-mCherry injected mice (Fig. 8*A–C*). The efficiency of ABC-*Creb1^KO^* might be underestimated because CREB was also expressed in the astrocytes that could not be targeted by neuron-specific Cre expression (Pardo et al., 2017). Consistent with previous studies (Graves et al., 2003; Wimmer et al., 2021), ABC-*Creb1^KO^* mice exhibited ∼100 min increase in daily NREMS amount, with no significant change in NREMS δ power (Fig. 8*D–K*). These results suggest that ABC-KO of *Creb1* can result in a significant sleep phenotype comparable with that of germline *Creb1* mutant mice (Graves et al., 2003; Wimmer et al., 2021).

### ABC-KO of exon 13 of *Sik3* phenocopies *Sleepy* mice

Forward genetic screening identified a *Sleepy* (*Sik3*^*Slp*/+^) mouse strain, in which a gain-of-function splicing mutation causes the skipping of exon 13 from *Sik3* transcripts and in-frame deletion of 52 amino acids from SIK3 proteins, an AMP-activated protein kinase (AMPK)-related protein kinase (Funato et al., 2016). The *Sik3*^*Slp*/+^ mice exhibit marked (∼250 min) increase of daily NREMS time accompanied with constitutively elevated NREMS δ power density, suggestive of inherently high sleep need (Funato et al., 2016). It should also be noted, however, that NREMS δ power does not equate sleep need, although it is often regarded as a good index of sleep need. Moreover, *Sik3* is an essential gene that is broadly expressed in mouse brain neurons (Funato et al., 2016), whereas *Sleepy* mutant mice also display other developmental phenotypes, such as obesity and reproductive defects (Funato H and Yanagisawa M, unpublished). Thus, it remains unclear whether the hypersomnia of *Sik3*^*Slp*/+^ mice is the primary phenotype owing to direct effects of SLP kinases in sleep regulation, or secondary phenotype resulted from the developmental defects of the brain or dysfunctions of peripheral organs.

**Figure 9.**
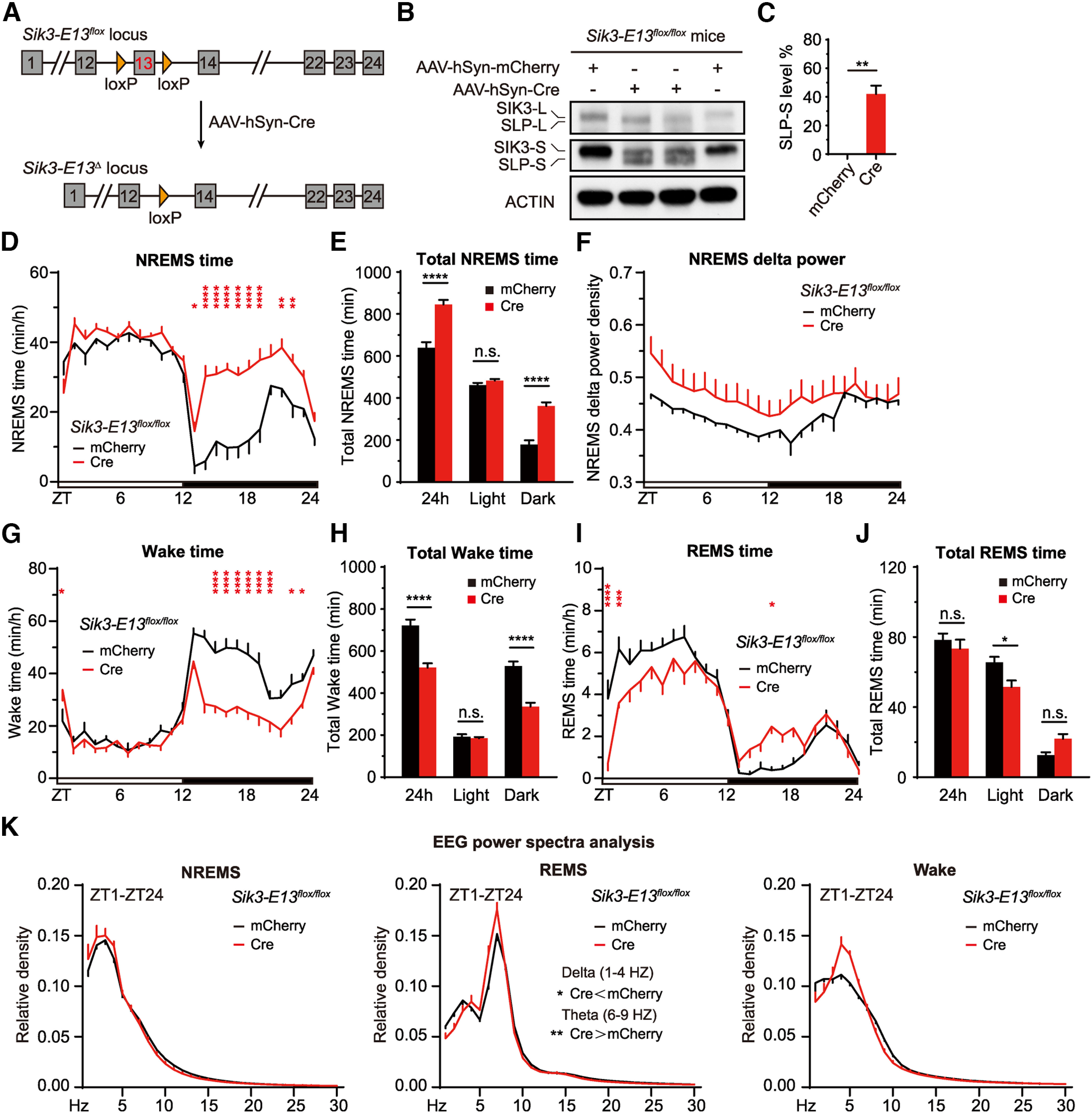
ABC-KO of exon 13 of *Sik3* by Cre-loxP recombination causes hypersomnia. ***A***, Schematic of ABC-KO of exon 13 of *Sik3* by AAV-hSyn-Cre injection of *Sik3-E13^flox/flox^* mice. ***B***, Immunoblotting of brain lysates from AAV-hSyn-mCherry or AAV-hSyn-Cre injected *Sik3-E13^flox/flox^* mice with anti-SIK3 and anti-ACTIN antibodies. ***C***, Quantification of SLP-S expression in ***B*** (*n* = 4), which is calculated by the percentage of SLP-S/(SIK3-S+SLP-S). ***D–F***, Hourly plot of NREMS time (***D***), quantification of total NREMS time (***E***), and hourly plot of NREMS δ power (***F***) in the AAV-hSyn-mCherry (*n* = 8) or AAV-hSyn-Cre (*n* = 8) injected *Sik3-E13^flox/flox^* mice. ***G–K***, Hourly plots of Wake (***G***) or REMS (***I***) time, quantification of total Wake (***H***) or REMS (***J***) time, and EEG power spectra analysis of NREMS, REMS, and Wake states (***K***) in the AAV-hSyn-mCherry (*n* = 8) or AAV-hSyn-Cre (*n* = 8) injected *Sik3-E13^flox/flox^* adult mice. Data are mean ± SEM. ***C***, Unpaired *t* test. ***D–K***, Two-way ANOVA with Dunn's multiple comparisons test. *p < 0.05; **p < 0.01; ***p < 0.001; ****p < 0.0001.

To distinguish among these possibilities, we performed retro-orbital injection of AAV-hSyn-Cre into *Sik3-E13^flox/flox^* adult mice, such that Cre/loxP-mediated recombination would convert *Sik3-E13^flox^* into a functionally equivalent *Slp* (*Sik3-E13*^Δ^) allele (Fig. 9*A*). Immunoblotting estimated that mutant SLP proteins were expressed in at least 40% of the adult brain neurons following AAV-hSyn-Cre injection (Fig. 9*B*,*C*). Accordingly, ABC-KO of exon 13 of *Sik3* induced marked hypersomnia, 200-300 min increase in daily NREMS time accompanied by constitutively elevated NREMS δ power, similar to that of *Sleepy* mice carrying the germline *Slp* mutation (Fig. 9*D–K*). These results suggest that the hypersomnia of *Sleepy* mice is the primary phenotype as a result of direct effects of mutant SLP kinases on the sleep regulatory machinery in the adult brain neurons.

### ABC-KO of genes by triple-target CRISPR in Cas9 mice

We reasoned that it would be more direct and faster to generate ABC-KO mice with the use of CRISPR/Cas9 technology. In this system, Cas9 nuclease is directed by single-guide (sg)RNA to introduce site-specific DNA break in the target gene, which is repaired by the error-prone nonhomologous end-joining pathways, resulting in indel mutations (e.g., short deletions or insertions) (Jinek et al., 2012; H. Wang et al., 2013; Hsu et al., 2014). However, these Cas9-mediated indel mutations occur at a moderate frequency, and not all mutations can ablate target gene function. Because the vast majority of adult brain neurons are nondividing, terminally differentiated cells, the efficiency of ABC-KO by CRISPR needs to be nearly 100%, such that both alleles of the target gene are disrupted in almost all AAV-transduced brain cells. Although it was recently reported that intravenous injection of AAV-PHP.eB-expressing single sgRNA can efficiently disrupt target gene in the majority of adult brain neurons (Xiao et al., 2021), this strategy often required extensive screening of sgRNAs and was not suitable for high-throughput analysis.

**Figure 10.**
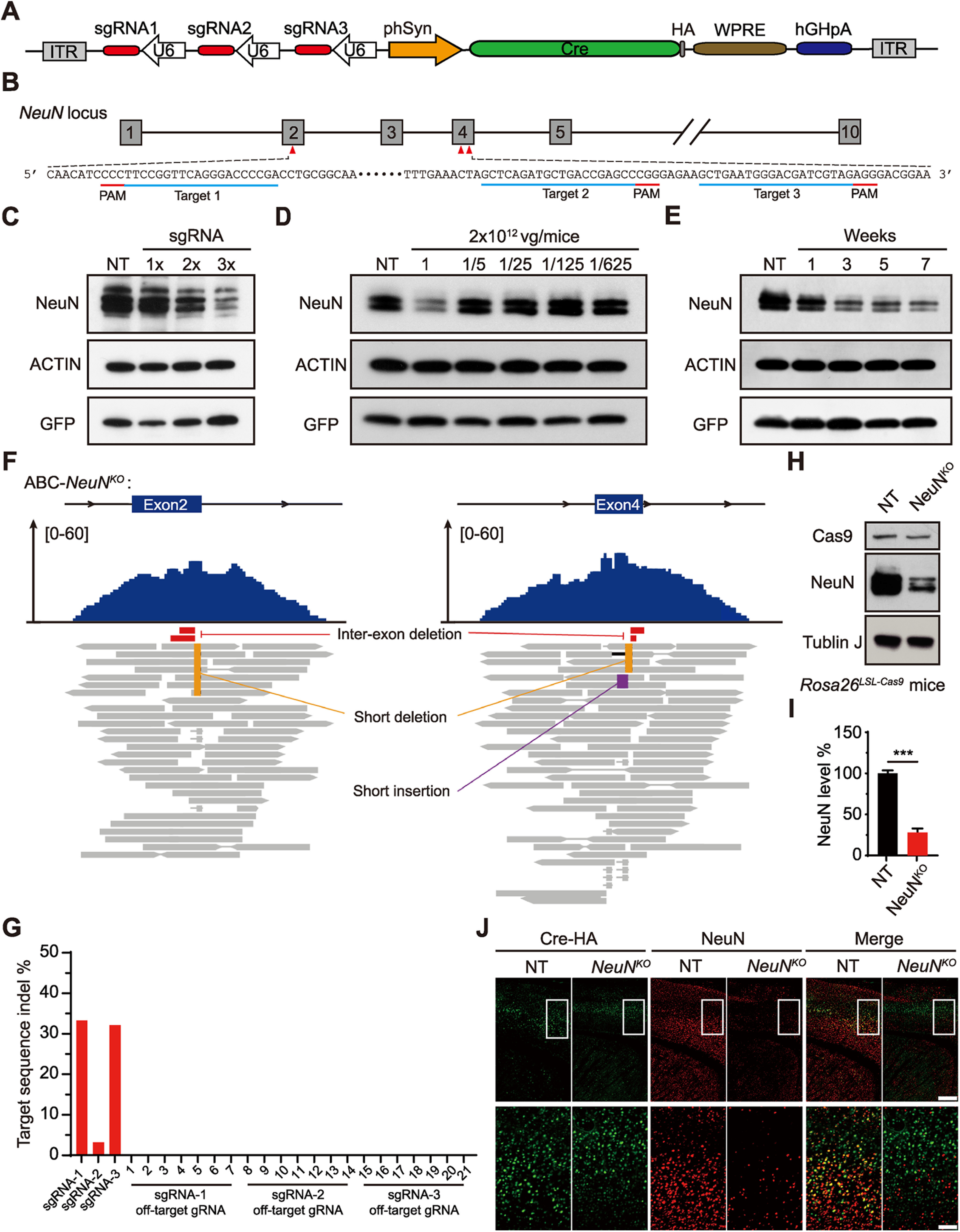
ABC-KO of target genes by triple-target CRISPR in Cas9 mice. ***A***, Schematic of AAV-3xsgRNA*^NeuN^* that expresses HA-Cre recombinase from hSyn promoter and three U6:sgRNA cistrons. ***B***, Schematic of target site sequences within exons 2 and 4 of *NeuN* gene. ***C***, Immunoblotting of NeuN proteins in brain lysates from AAV-3xsgRNA^NT^, AAV-1xsgRNA*^NeuN^*, AAV-2xsgRNA*^NeuN^*, AAV-3xsgRNA*^NeuN^* (10^12^ vg/mice) injected *Rosa26^LSL-Cas9^* mice. ***D***, Immunoblotting of NeuN proteins in brain lysates from Cas9 mice injected with different doses of AAV-3xsgRNA*^NeuN^*. ***E***, Immunoblotting of NeuN proteins in brain lysates from AAV-3xsgRNA*^NeuN^* injected Cas9 mice at 1, 3, 5, or 7 weeks after (10^12^ vg/mice) virus injection. AAV-3xsgRNA^NT^ injected mouse brains were collected at 3 weeks after virus injection. ***F***, Genomic alignments of whole genome sequencing reads of ABC-*NeuN^KO^* mouse brain DNA at the target sites within exons 2 and 4 of *NeuN* gene. Top and bottom panels represent read coverage and read alignments, respectively, with different types of mutations highlighted. ***G***, Quantitation of indel mutations at the 3 target sites and 21 predicted off-target sites for the three sgRNAs targeting *NeuN* gene based on the whole genome sequencing data. ***H***, Immunoblotting of whole-brain lysates from AAV-3xsgRNA^NT^ and AAV-3xsgRNA*^NeuN^* injected *Rosa26^LSL-Cas9^* mice with the corresponding antibodies. ***I***, Quantification of the level of NeuN expression in ***H*** (*n* = 4). ***J***, Coimmunostaining of HA-Cre and NeuN in the PFC sections of AAV-3xsgRNA^NT^ and AAV-3xsgRNA*^NeuN^* injected mice. Bottom row represents magnified images of the corresponding boxed regions in the top row. Scale bars: top, 400 μm; bottom, 100 μm. Data are mean ± SEM. ***I***, Unpaired *t* test. *p < 0.05; **p < 0.01; ***p < 0.001; ****p < 0.0001.

Multiplexing strategies using several sgRNAs targeting the same gene have been used to improve the efficiency of KO by CRISPR in various model organisms (Xie et al., 2015; Yin et al., 2015; Port et al., 2020). Notably, triple-target CRISPR in zygotes can produce whole-body biallelic KO mice with 96%-100% efficiency in a single generation (Sunagawa et al., 2016; Tatsuki et al., 2016). Thus, we compared the efficiency of ABC-KO by CRISPR by injecting *Rosa26^LSL-Cas9^* mice with AAV-PHP.eB-expressing HA-tagged Cre recombinase from the hSyn promoter as well as 1, 2, or 3 U6:sgRNA cistrons targeting *NeuN*, a ubiquitously expressed gene in the adult brain neurons (Fig. 10*A*,*B*). The efficiency of ABC-KO of *NeuN* was significantly higher with triple sgRNAs than with single or double sgRNAs (Fig. 10*C*). Moreover, the efficiency of ABC-*NeuN^KO^* increased in a viral dose-dependent manner and peaked at 3 weeks after AAV injection (Fig. 10*D*,*E*). Whole genome sequencing of ABC-*NeuN^KO^* mouse brain revealed on-target indel mutations and large inter-exon deletions, but rarely off-target mutations (Fig. 10*F*,*G*). Accordingly, immunoblotting showed that the level of NeuN expression was specifically reduced by ∼70%, whereas another pan-neuronal protein Tublin J remain unchanged in the ABC-*NeuN^KO^* brain lysates (Fig. 10*H*,*I*). Coimmunostaining of NeuN and Cre indicated that the expression of NeuN disappeared, indicative of biallelic KO of *NeuN*, in the majority of the adult brain neurons expressing both Cre and 3xsgRNA^NeuN^ (Fig. 10*J*).

### ABC-CRISPR of *slp/Sik3* rescues hypersomnia of *Sik3*^*Slp*/+^ mice

**Figure 11.**
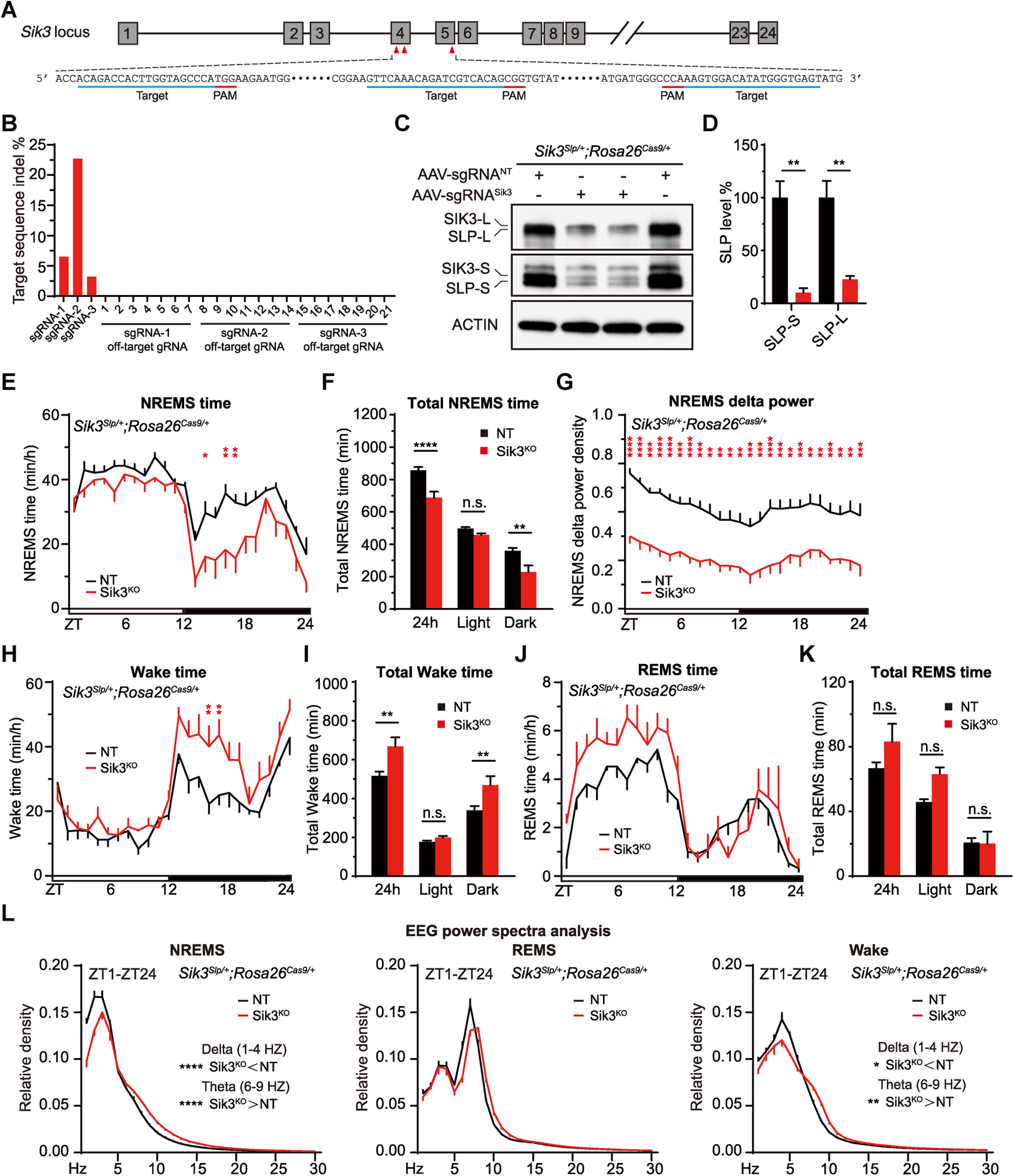
ABC-CRISPR of *Slp/Sik3* rescues hypersomnia of *Sik3*^*Slp*/+^*;Rosa26*^*Cas9*/+^ mice. ***A***, Schematic showing triple sgRNA target sites within exons 3, 4, and 5 of *Sik3* gene. ***B***, Quantitative analysis of indel mutations at the sgRNA target sites and 21 predicted off-target sites for the three sgRNAs targeting *Sik3* gene. ***C***, Immunoblotting of whole-brain lysates from AAV-sgRNA^NT^ and AAV-sgRNA*^Sik3^* injected *Sik3*^*Slp*/+^*; Rosa26*^*Cas9*/+^ mice with anti-SIK3 and anti-ACTIN antibodies. ***D***, Quantification of the levels of SIK3-L/SLP-L and SIK3-S/SLP-S proteins shown in ***C*** (*n* = 3). ***E–G***, Hourly plot of NREMS time (***E***), quantification of total NREMS time (***F***), and hourly plot of NREMS δ power (***G***) in the AAV-sgRNA^NT^ (*n* = 7) or AAV-sgRNA*^Sik3^* (*n* = 7) injected *Sik3*^*Slp*/+^; *Rosa26*^*Cas9*/+^ mice. ***H–L***, Hourly plots of Wake (***H***) or REMS (***J***) time, quantification of total Wake (***I***) or REMS (***K***) time, and EEG power spectra analysis of NREMS, REMS, and Wake states (***L***) in the AAV-sgRNA^NT^ (*n* = 7) or AAV-sgRNA*^Sik3^* (*n* = 7) injected *Sik3*^*Slp*/+^; *Rosa26*^*Cas9*/+^ mice. Data are mean ± SEM. ***D***, Unpaired *t* test. ***E–L***, Two-way ANOVA with Dunn's multiple comparisons test. *p < 0.05; **p < 0.01; ***p < 0.001; ****p < 0.0001.

Because ABC-KO of exon 13 of *Sik3* could induce hypersomnia in adult mice (Fig. 9*D*,*E*), we hypothesized that ABC-KO of *Slp/Sik3* by triple-target CRISPR should rescue hypersomnia of *Sik3*^*Slp*/+^ mice. To test our hypothesis, we performed ABC-CRISPR of *Slp/Sik3* alleles by injecting constitutively Cas9-expressing *Sik3*^*Slp*/+^;*Rosa26*^*Cas9*/+^ adult mice with AAV-3xsgRNA*^Sik3^* expressing triple sgRNAs targeting different exons of *Sik3* gene (Fig. 11*A*). Whole genome sequencing and genomic PCR revealed both on-target indel mutations and large inter-exon deletions between distinct sgRNA target sites, but rarely off-target mutations, in the AAV-3xsgRNA^Sik3^-injected mouse brains (Fig. 11*B*). As shown by Western blotting, the expression level of SIK3/SLP proteins were estimated to reduce by ∼75% in the ABC-*Sik3^KO^* brain lysates (Fig. 11*C*,*D*). Importantly, ABC-CRISPR of *Slp/Sik3* resulted in ∼150 min reduction in daily NREMS time accompanied by constitutively reduced NREMS δ power in *Sik3*^*Slp*/+^;*Rosa26*^*Cas9*/+^ male mice (Fig. 11*E–L*). Likewise, ABC-CRISPR of *Slp/Sik3* using a second set of sgRNAs caused ∼180 min reduction in daily NREMS time with diminished NREMS δ power in *Sik3*^*Slp*/+^;*Rosa26*^*Cas9*/+^ female mice (Liu Q, unpublished). These results suggest that the hypersomnia of *Sleepy* mice requires continuous expression of mutant SLP kinases in the adult brain neurons.

### Multiplex ABC-CRISPR of orexin/hypocretin receptors causes narcolepsy-like episodes

**Figure 12.**
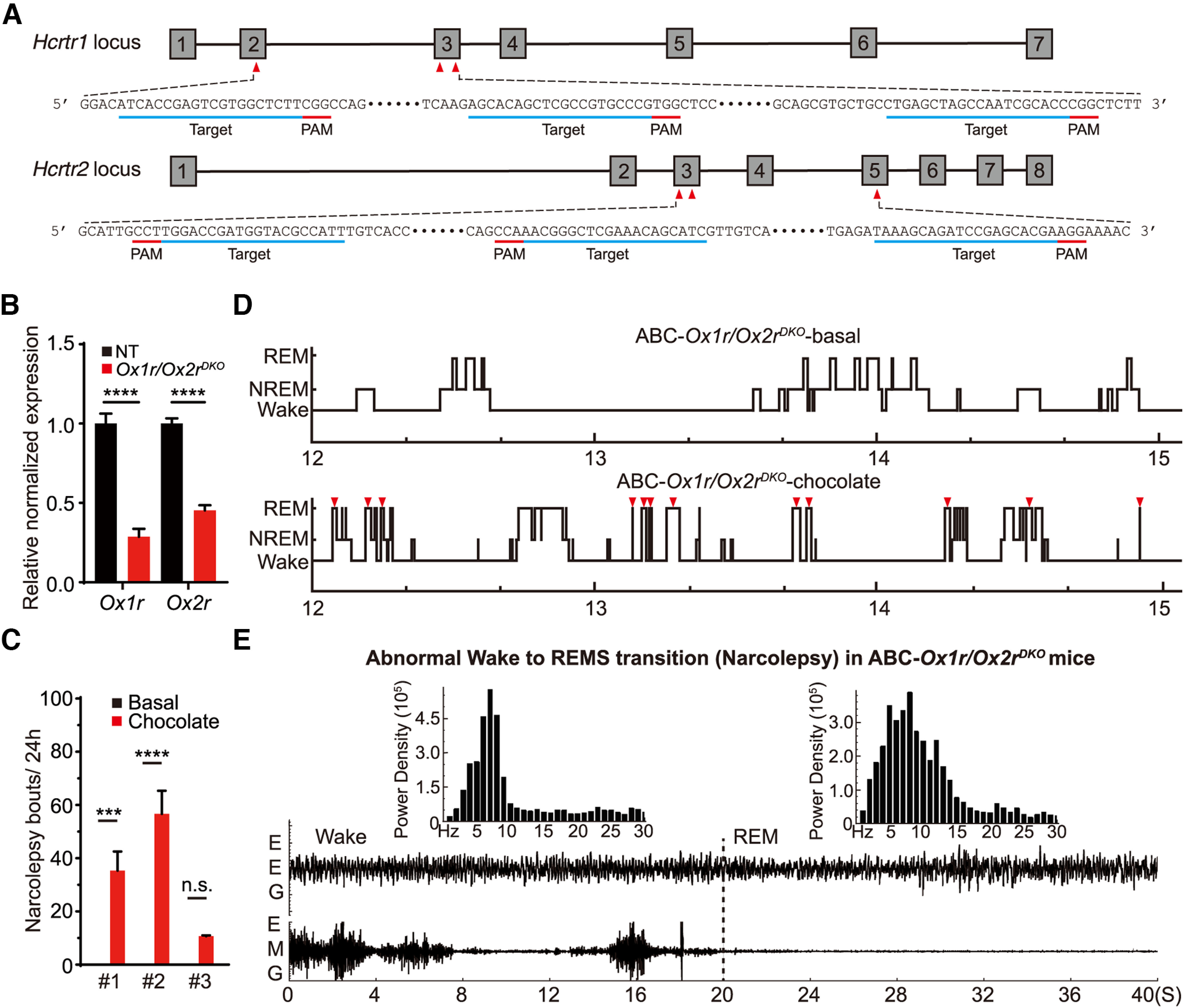
Multiplex ABC-CRISPR of *Ox1r* and *Ox2r* causes chocolate-induced narcolepsy episodes. ***A***, Schematic showing the two sets of sgRNA target sites within exons 2 and 3 of *Ox1r* gene and exons 3 and 5 of *Ox2r* gene. ***B***, Quantification of *Ox1r* and *Ox2r* transcript levels in the ABC-NT and ABC-*Ox1r/Ox2r^DKO^* mouse brains by real-time qRT-PCR. ***C***, Quantification of the number of narcolepsy episodes in 3 ABC-*Ox1r/Ox2r^DKO^* mice before and after chocolate feeding for 3 d. ***D***, Representative hypnograms (ZT12-15) of ABC-*Ox1r/Ox2r^DKO^* mice before and after chocolate feeding. Red triangles represent abnormal wake to REM transitions during narcolepsy-like episodes. ***E***, Representative EEG/EMG signals depicting the abnormal wake to REM transition during one narcoleptic episode in the ABC-*Ox1r/Ox2r^DKO^* mice. Data are mean ± SEM. ***B***, ***C***, *p < 0.05; **p < 0.01; ***p < 0.001; ****p < 0.0001; unpaired *t* test.

The deficiency of neuropeptide orexin/hypocretin or its receptors, OX1R/HCRTR1 and OX2R/HCRTR2 (hereafter OX1R and OX2R for simplicity), results in narcolepsy-like phenotypes, such as abnormal wake to REMS transition and cataplexy, in mice (Chemelli et al., 1999; Kalogiannis et al., 2010; Kohlmeier et al., 2013). For double ABC-CRISPR of OX1R and OX2R, we coinjected Cas9-expressing mice with two AAV-PHP.eB viruses expressing separate sets of triple sgRNAs targeting either *Ox1r* or *Ox2r* gene, respectively (Fig. 12*A*). As shown by qRT-PCR, the levels of *Ox1r* and *Ox2r* transcripts were reduced by ∼75% and ∼60%, respectively, in the AAV-sgRNA*^Ox1r/Ox2r^*-injected (ABC-*Ox1r/Ox2r^DKO^*) relative to AAV-sgRNA^NT^-injected (ABC-NT) mouse brains (Fig. 12*B*). At first, all of the ABC-*Ox1r/Ox2r^DKO^* mice exhibited no sleep abnormality compared with the ABC-NT control mice. After feeding with chocolates, a known stimulant of narcolepsy (Oishi et al., 2013), 3 of 8 ABC-*Ox1r/Ox2r^DKO^* mice exhibited frequent narcolepsy-like episodes, which was characterized by the abnormal transitions from wake to REMS during EEG/EMG recording (Fig. 12*C–E*). However, we did not observe cataplexy during the narcolepsy episodes through simultaneous video/EEG recording and manual inspection. This partial narcoleptic phenotype was probably because of incomplete KO of OX1R and OX2R in the adult brain neurons. These results suggest that multiplex ABC-CRISPR can enable one-step analysis of the sleep phenotypes of redundant genes in adult mice.

## Discussion

### High-throughput somatic genetics screening of sleep regulatory genes in adult mice

**Figure 13.**
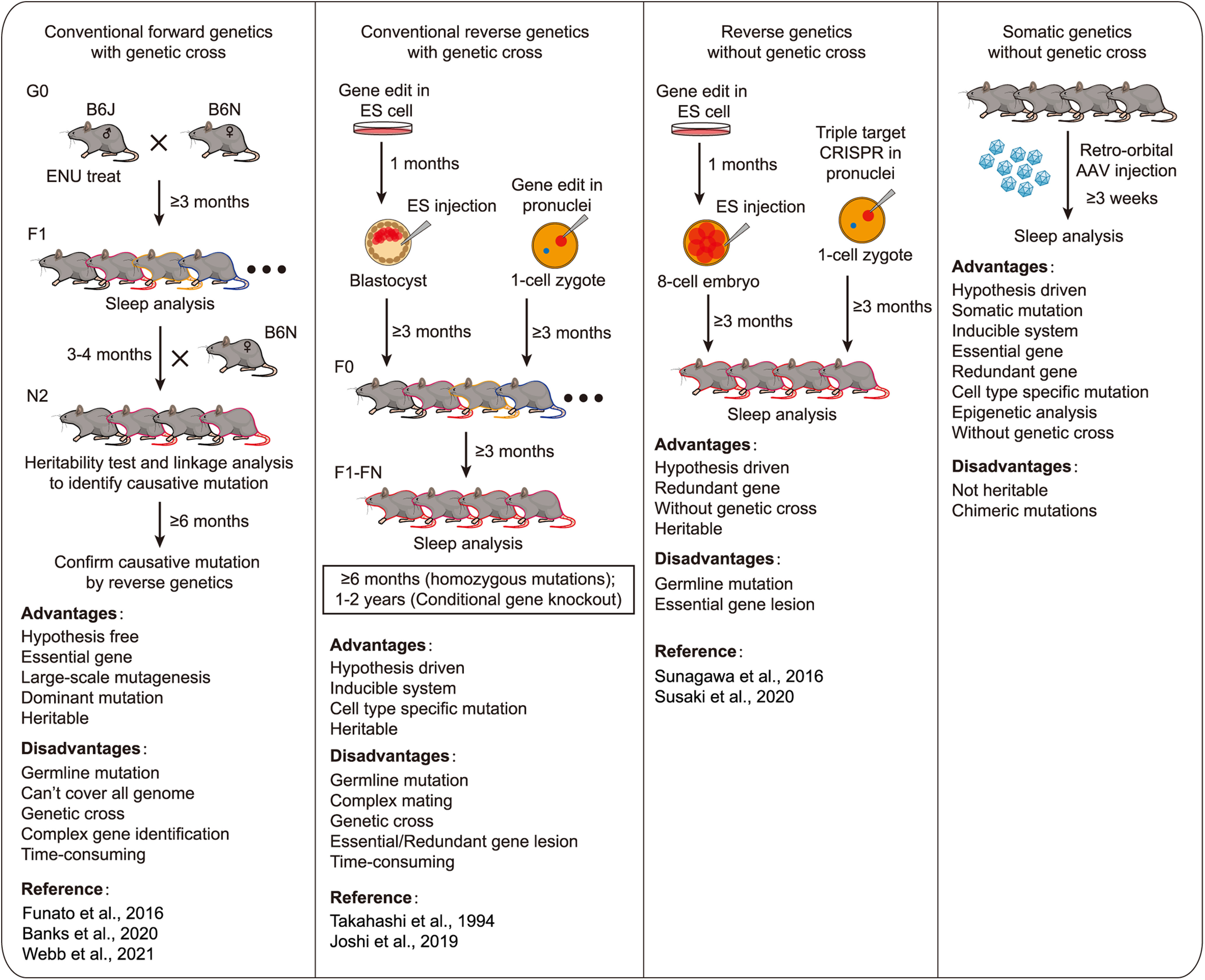
Comparison between forward/reverse genetics and somatic genetics approaches in mouse sleep study. The detailed procedures and advantages/disadvantages of forward, reverse, and somatic genetics approaches for mouse sleep analysis.

The molecular mechanisms of mammalian sleep regulation remain largely unknown. Forward genetics screening of randomly mutagenized mice is a powerful and hypothesis-free approach to identify key sleep regulatory genes in mammals (Takahashi et al., 1994; Kapfhamer et al., 2002; Funato et al., 2016; Banks et al., 2020). On the other hand, reverse genetics, through the making of transgenic, knockin, KO, and conditional KO mice for specific genes of interest, represents a hypothesis-driven approach to identify and characterize new sleep regulatory genes (Takahashi et al., 1994; Graves et al., 2003; Hellman et al., 2010; Mikhail et al., 2017; Honda et al., 2018). This latter approach is greatly accelerated by next-generation gene-editing technologies, such as the CRISPR/Cas9 system (H. Wang et al., 2013; Hsu et al., 2014; Sunagawa et al., 2016; Tatsuki et al., 2016). However, classical forward and reverse genetics approaches involve germline mutations and, therefore, are unwieldy to study the essential genes (owing to early lethality) or redundant genes (owing to lack of phenotype) (Fig. 13).

In this study, we described the AAV-mediated ABC-expression/KO platform for rapid and efficient somatic genetics analysis of sleep phenotypes in adult mice (Fig. 13). We conducted a pilot ABC-expression sleep screen that identified CREB and a new sleep regulator CRTC1. Both constitutive and inducible expression of CREB and CRTC1 in the adult brain neurons significantly reduced daily NREMS amount. There are two well-known mechanisms for activation of CREB activity: (1) phosphorylation of S133 of CREB; and (2) CRTCs function as coactivators for CREB. Our results suggest that the ability of CREB to regulate sleep can be enhanced by CRTCs but is probably independent of S133 phosphorylation. ABC-expression of CRTC1, but not CREB, also reduced NREMS δ power, suggesting that specific target genes of CRTC1 may regulate sleep intensity. These results demonstrate the proof of principle that somatic genetics screening can facilitate rapid identification of new sleep regulatory genes, including essential or redundant genes, in adult mice.

We also developed a highly accurate, noninvasive, and automated SleepV (video) system for high-throughput mouse sleep screening to identify core sleep regulators that significantly impact daily sleep time. SleepV has several improvements compared with previously published video-based systems for sleep/wake staging (Pack et al., 2007; Fisher et al., 2012; Banks et al., 2020). First, SleepV uses a pretrained deep neural network to identify the mouse from background. Second, SleepV uses multiple parameters, including predict, movement, mask area, and color, to judge whether the mouse is active or not. Third, SleepV annotates ≤15 s of mouse movements in between two sleep states as sleep. All of these improvements contribute to the high (∼96.4%) accuracy of SleepV in sleep/wake staging, which is better than the (92%-94%) accuracy of existing video-based systems. As a result of this small yet significant improvement, the accuracy of fully automatic SleepV is comparable to that of semiautomatic EEG/EMG analysis with manual corrections, making SleepV more suitable for high-throughput mouse sleep screening. It should also be noted that this video-based approach has some inherent limitations, such as inability to detect REMS or EEG abnormalities and occasionally underestimating sleep owing to muscle twitching during sleep or overestimating sleep if mice move much less when awake.

### ABC-KO by AAV-Cre injection facilitates systematic screening of conditional flox mice

To bypass embryonic lethality, conditional flox mice are sometimes crossed with CaMKIIα-Cre transgenic mice, which begin to express Cre recombinase postnatally (Tsien et al., 1996). Alternatively, conditional flox mice can be crossed with transgenic mice expressing from a pan-neuronal promoter the Cre^ERT2^ fusion protein between Cre and mutant estrogen receptor, which becomes activated and translocates to the nucleus on binding of synthetic 4-hydroxytamoxifen (Feil et al., 1996). However, both methods have significant drawbacks: (1) CaMKIIα-Cre mice express Cre only in the excitatory neurons of the forebrain and Cre expression begins days after birth; and (2) Cre^ERT2^-mediated loxP recombination is inefficient after tamoxifen injection in adult mice (Iwasaki et al., 2021).

We showed that intravenous injection of *Creb1^flox/flox^* adult mice with AAV-hSyn-Cre could efficiently KO CREB expression in the adult brain neurons and significantly increase daily NREMS amount. Moreover, AAV-hSyn-Cre injection efficiently excised exon 13 of *Sik3* in the adult brain neurons of *Sik3-E13^flox/flox^* mice, which phenocopied germline *Sleepy* mutant mice. Thus, AAV-mediated Cre/loxP recombination can create either loss- or gain-of-function somatic mutations of target gene in the mouse brain for sleep phenotype analysis. Notably, this simple method of ABC-KO of exon 13 of *Sik3* by AAV-Cre injection of *Sik3-E13^flox/flox^* adult mice is much more efficient than the traditional method of breeding *synapsin 1-Cre^ERT2^;Sik3-E13*^*flox*/+^ mice, which exhibit little or no sleep phenotype after tamoxifen injection at the adult stage (Iwasaki et al., 2021).

A recent development for mouse genetics is the availability of a large repertoire of conditional flox strains for most mouse genes from the International Knockout Mouse Consortium and commercial sources. Therefore, we envision that ABC-KO by AAV-Cre injection will facilitate systematic screening of conditional flox mice for sleep and other brain-related phenotypes. Moreover, AAV-delivered Cre expression from various promoters, such as the hSyn promoter (neurons), CaMKII promoter (excitatory neurons), or mDlx promoter (inhibitory neurons) (Tsien et al., 1996; de Leeuw et al., 2016; Dimidschstein et al., 2016; Graybuck et al., 2021; Mich et al., 2021), represents a simple and efficient method to further investigate cell type-specific functions of target gene in the mouse brain without crossing with various Cre transgenic mice.

### Multiplex ABC-CRISPR enables one-step analysis of redundant sleep genes

To analyze sleep phenotype of redundant genes, it is time-consuming (≥2 years) to generate double or triple KO mice through classical germline genetic approaches (Sunagawa et al., 2016). A combination of triple-target CRISPR and modified embryonic stem cell technologies allows for biallelic KO of multiple nonessential genes in a single generation for sleep phenotype analysis (Sunagawa et al., 2016). Here, we successfully used multiplex ABC-CRISPR technology to disrupt both *Ox1r* and *Ox2r* genes in adult mice, resulting in chocolate-induced narcolepsy episodes. It is worth noting that the efficiency of multiplex ABC-CRISPR can be further improved by optimizing sgRNA structure or prescreening of sgRNAs, and by potentially developing other CRISPR/Cas systems. For example, unlike CRISPR/Cas9 that requires three U6:sgRNA cistrons to target the same gene, CRISPR/Cpf1 (Cas12a) and CRISPR/Cas13d (targeting RNA) can process a polycistronic transcript into multiple gRNAs targeting the same or different genes (Zetsche et al., 2017; Zhong et al., 2017; Konermann et al., 2018). Thus, multiplex ABC-CRISPR will enable one-step analysis of redundant sleep genes in adult mice, which is also applicable for essential genes and can be achieved within 1-2 months.

### How to distinguish between primary versus secondary sleep phenotypes

A large number of genes play important roles in the development of mouse brain. Thus, it is often uncertain whether the sleep phenotype of a mutant mouse strain is the primary phenotype or secondary phenotype owing to developmental abnormalities of the brain. The ABC-expression/KO platform can be used to effectively address this challenging question by skipping mouse development and directly assessing the sleep phenotypes of somatic mutations in the adult brain neurons. For example, ABC-KO of exon 13 of *Sik3* induced hypersomnia in *Sik3-E13^flox/flox^* mice, whereas ABC-CRISPR of *Slp/Sik3* largely reversed hypersomnia in *Sleepy* (*Sik3*^*Slp*/+^) mice. These results strongly suggest that the hypersomnia of *Sleepy* mice is the primary phenotype owing to a direct role of mutant SLP kinase in sleep regulation in the adult brain neurons. Thus, this type of somatic genetics analysis can serve as an effective tool to distinguish between the primary and secondary sleep phenotypes for genes that are also important for mouse brain development.

### Somatic genetics analysis of the molecular pathways of sleep regulation

The ABC-expression/KO platform is also highly efficient for conducting sophisticated genetics experiments in adult mice by minimizing genetic crosses. When ABC-expression/KO of two different genes produce opposite sleep phenotypes, epistasis analysis can be rapidly conducted to determine whether the two genes operate in the same or parallel pathways and map the order of these genes if they are in the same pathway. A potential caveat is that sometimes epistasis analysis is difficult to interpret in the setting of mosaic or non-null mutations. For structural and functional analysis of key sleep regulators, ABC-expression of WT and mutant proteins can be conducted to rescue the sleep phenotypes of ABC-KO of endogenous proteins. To identify downstream effectors of SLP/SIK3 kinases in sleep regulation, a suppressor screen can be easily conducted by ABC-expression of candidate proteins in *Sik3*^*Slp*/+^ mice or by ABC-CRISPR of candidate genes in *Sik3*^*Slp*/+^;*Rosa26*^*Cas9*/+^ mice to rescue the *Sleepy* phenotype. Conversely, the ABC-expression/KO platform can be used for enhancer screens to uncover redundant pathways of sleep regulation. Thus, this AAV-based somatic genetics approach should facilitate not only rapid identification of new sleep regulatory genes, but also efficient characterization of the molecular pathways of sleep regulation in mice. Finally, we envision that this somatic genetics approach may also be useful for studies of other brain-related physiological and behavioral processes.
